# Long-term proteasomal inhibition in transgenic mice by UBB^+1^ expression results in dysfunction of central respiration control reminiscent of brainstem neuropathology in Alzheimer patients

**DOI:** 10.1007/s00401-012-1003-7

**Published:** 2012-06-23

**Authors:** Martin Irmler, Romina J. G. Gentier, Frank J. A. Dennissen, Holger Schulz, Ines Bolle, Sabine M. Hölter, Magdalena Kallnik, Jing Jun Cheng, Martin Klingenspor, Jan Rozman, Nicole Ehrhardt, Denise J. H. P. Hermes, Valérie Gailus-Durner, Helmut Fuchs, Martin Hrabě de Angelis, Helmut E. Meyer, David A. Hopkins, Fred W. Van Leeuwen, Johannes Beckers

**Affiliations:** 1 Helmholtz Zentrum München, National Research Center for Environment and Health, GmbH, Institute of Experimental Genetics, Ingolstaedter Landstr. 1, 85764 Neuherberg, Germany; 2 Department of Neuroscience, Faculty of Health, Medicine and Life Sciences, Maastricht University, Universiteitssingel 50, 6229 ER Maastricht, The Netherlands; 3 Helmholtz Zentrum München, National Research Center for Environment and Health, GmbH, Institute of Lung Biology and Disease, Ingolstaedter Landstr. 1, 85764 Neuherberg, Germany; 4 Helmholtz Zentrum München, National Research Center for Environment and Health, GmbH, Institute of Developmental Genetics, Ingolstaedter Landstr. 1, 85764 Neuherberg, Germany; 5 Technische Universität München, ZIEL—Research Center for Nutrition and Food Sciences, Molecular Nutritional Medicine, Gregor-Mendel-Straße 2, 85350 Freising-Weihenstephan, Germany; 6 Technische Universität München, WZW—Center of Life and Food Science Weihenstephan, Chair of Experimental Genetics, 85350 Freising-Weihenstephan, Germany; 7 Helmholtz Zentrum München, National Research Center for Environment and Health, GmbH, Institute of Epidemiology I, Ingolstaedter Landstr. 1, 85764 Neuherberg, Germany; 8 Medical Proteome Center, Ruhr University Bochum, Bochum, Germany

**Keywords:** Alzheimer’s disease (AD), Mouse model, Ubiquitin B (UBB^+1^), Ubiquitin–proteasome system (UPS), Hypoxic response, Central breathing control

## Abstract

Aging and neurodegeneration are often accompanied by a functionally impaired ubiquitin–proteasome system (UPS). In tauopathies and polyglutamine diseases, a mutant form of ubiquitin B (UBB^+1^) accumulates in disease-specific aggregates. UBB^+1^ mRNA is generated at low levels in vivo during transcription from the ubiquitin B locus by molecular misreading. The resulting mutant protein has been shown to inhibit proteasome function. To elucidate causative effects and neuropathological consequences of UBB^+1^ accumulation, we used a UBB^+1^ expressing transgenic mouse line that models UPS inhibition in neurons and exhibits behavioral phenotypes reminiscent of Alzheimer’s disease (AD). In order to reveal affected organs and functions, young and aged UBB^+1^ transgenic mice were comprehensively phenotyped for more than 240 parameters. This revealed unexpected changes in spontaneous breathing patterns and an altered response to hypoxic conditions. Our findings point to a central dysfunction of respiratory regulation in transgenic mice in comparison to wild-type littermate mice. Accordingly, UBB^+1^ was strongly expressed in brainstem regions of transgenic mice controlling respiration. These regions included, e.g., the medial part of the nucleus of the tractus solitarius and the lateral subdivisions of the parabrachial nucleus. In addition, UBB^+1^ was also strongly expressed in these anatomical structures of AD patients (Braak stage #6) and was not expressed in non-demented controls. We conclude that long-term UPS inhibition due to UBB^+1^ expression causes central breathing dysfunction in a transgenic mouse model of AD. The UBB^+1^ expression pattern in humans is consistent with the contribution of bronchopneumonia as a cause of death in AD patients.

**Electronic supplementary material:**

The online version of this article (doi:10.1007/s00401-012-1003-7) contains supplementary material, which is available to authorized users.

## Introduction

In eukaryotic cells, the function of complex protein networks is maintained by highly conserved pathways that regulate the selective degradation of proteins in a spatially and temporally organized manner. For example, the ubiquitin–proteasome system (UPS) contributes considerably to protein quality control together with the autophagy-lysosomal system. The UPS relies on the tagging of proteins destined for breakdown by the attachment of polyubiquitin chains and the subsequent translocation and degradation by proteasomes. The coupling of polyubiquitin chains to proteins is mediated by a cascade of E1, E2 and E3 proteins [21], where specificity is brought about by a large number of E3 ligases (>600). The complexity of the UPS is further increased by various deubiquitination enzymes (>90), the length and linkage type of the polyubiquitin chains, as well as by several ubiquitin-like proteins. In neuronal cells, the UPS is required for both pre- and postsynaptic processes [12, 37]. As a consequence, axonal outgrowth, long-term potentiation, and memory formation are critically dependent on a functional UPS [43].

Mutations in UPS genes have been associated with the pathogenesis of neurodegenerative and other brain diseases. For example, defects in the E3 ligase Park2 are causative for familiar forms of Parkinson’s disease (PD) [26]. Loss-of-function alleles of the deubiquitinase ubiquitin C-terminal hydrolase (UCHL1) are associated with the two most prevalent neurodegenerative diseases, PD and AD [34]. Both diseases are also characterized by the presence of ubiquitin (Ub) in disease-specific aggregates [30] and by a partial impairment of the UPS in general [18, 25], as is also observed in the elderly where age-related neurodegeneration is most prevalent. Despite the strong correlation between the UPS, Ub-containing cellular deposits and neurodegeneration, little is known about the impact of UPS inhibition on disease onset or progression. However, the presence of a mutant form of ubiquitin B, the UBB^+1^ protein, in disease-specific aggregates (e.g., neurofibrillary tangles and intranuclear inclusions) of patients suffering from tauopathies and polyglutamine diseases [7, 10] may point to a causative role of UPS dysfunction in these diseases rather than being a consequence. UBB^+1^ mRNA is formed at a low level [17] during transcription from the ubiquitin B (UBB) locus by a process called “molecular misreading” [38]. Dinucleotide deletions (ΔGU) in the *Ubb* transcripts occur just next to a GAGAG motif and result in a +1 reading frame shift and an altered protein sequence [39]. The resulting UBB^+1^ protein has a 20-residue extension and lacks the C-terminal glycine residue required for conjugation to target proteins for degradation. In the elderly, UBB^+1^ protein levels are known to increase. At higher concentrations, UBB^+1^ is known to inhibit the proteasome [42] and may therefore contribute to disease progression. In line with these results, UBB^+1^ accumulates in the neuropathological hallmarks of all types of AD patients [39–41]. UBB^+1^ transgenic (tg) mice (line 3413) overexpress human UBB^+1^ specifically in neurons of the postnatal brain, with strongest neuronal expression in the forebrain [11]. In keeping with this, these mice have spatial and contextual memory deficits [11]. UBB^+1^ tg mice are currently the only mammalian in vivo model for long-term proteasome inhibition in the brain. However, in contrast to AD patients, no overt neuronal atrophy or degeneration has been observed in these mice. The expression of cerebral cortical proteins implicated in energy metabolism was altered, thus resembling AD in some aspects [11]. Consequently, as the central nervous system is a major regulator of body function, UBB^+1^ expression in brain might also indirectly affect the functioning of other organ systems. To further explore this, we extended our previous analysis of UBB^+1^ tg mice by a systematic phenotyping analysis in the German Mouse Clinic, which has been successfully used to discover novel and additional phenotypes [2, 9]. Most interestingly, this approach revealed a respiratory dysfunction that was mirrored by transgenic UBB^+1^ expression in brainstem nuclei involved in breathing control. A similar distribution of UBB^+1^ expression was also observed in the same brain areas of AD patients. In addition to hippocampal transgene expression in line 3413, we found significant but mild changes in behavior and metabolic parameters of UBB^+1^ transgenic mice, which may be additional consequences of UBB^+1^ expression in brainstem areas [19]. These data are consistent with human anatomical data reporting that tangle formation appears early in subcortical brain areas such as the locus coeruleus [4, 5, 31].

## Materials and methods

### Animal model and systematic phenotyping

UBB^+1^ overexpressing transgenic mice [UBB^+1^ tg, line 3413, 008833C57Bl/6.Tg(CaMK2α-UBB) 3413, Jackson #008833] [11] were kept on a C57BL/6J background and heterozygous transgenic mice were analyzed further. Non-transgenic littermates were used as controls. To ensure comparability among individuals, all mice were kept under standard animal housing conditions [e.g., bedding, specified pathogen free (SPF) conditions, light/dark cycle, and climate control] with food and water ad libitum. Mice were analyzed according to the primary systemic phenotyping approach in the German Mouse Clinic at two different ages of approximately 3 and 12 months as described below and elsewhere [16]. Secondary phenotyping screens to extend our primary findings were carried out at additional ages as described in the manuscript. Respiratory functions are presented in detail because these were the most significant effects identified by the primary screening protocol.

### Respiratory function

Spontaneous breathing patterns of male and female mice (UBB^+1^ tg mice and controls) were studied at the ages of 3 (*n* = 6 for all four groups), 12 (*n* = 6 for all four groups) and 18 months (*n* = 8 for male tg mice and controls; females were not analysed). For the analysis of lung function and of the ventilatory response to hypercapnic and hypoxic conditions, males at the age of 18 months were studied (*n* = 8). A detailed description of the whole body plethysmography and the assessment of the lung function are provided in the supplementary methods. Statistical analyses were performed using a commercially available statistics package (Statgraphics^®^, Statistical Graphics Corporation, Rockville, MD, USA). Differences between groups were evaluated by Student’s *t* test. Statistical significance was assumed at *p* < 0.05. Data are presented as mean ± standard error of the mean (SEM).

### Immunohistochemistry of mouse and human tissues

Eight 3-month-old, four 6-month-old, five 15-month-old male and four 18-month-old UBB^+1^ tg mice and three 3-month-old and three 15-month-old male control mice were given deep pentobarbital anesthesia (i.p.) and were perfused intracardially with phosphate-buffered saline (PBS) followed by PBS containing 4 % (w/v) paraformaldehyde (pH 7.4). Dissected brains were sectioned either coronally or sagittally in 50 μm thick sections with a Vibratome. Sections were stained with anti-UBB^+1^ antibody (Ubi3 16/09/97, final dilution 1:1,000) [10]. As controls for Ubi3 immunoreactivity, the Ubi3 antiserum was adsorbed with ^6^His tag UBB^+1^ and the pre-immune control serum was used (other controls were reported previously, [11]). In addition, an anti-14-3-3 zeta antibody (ζ, #1001) was generously supplied by A. Aitken (Edinburgh, UK) and tested on brain sections of UBB^+1^ tg mice (final dilution 1:500). After the primary antibody incubation, the sections were incubated with a biotinylated donkey anti-rabbit antibody followed by avidin–biotin–peroxidase and 3,3′-diaminobenzidine (DAB) intensified by 0.2 % nickel ammonium sulphate (pH 7.6). Photographs were made using the dotSlide BX51 microscope (Olympus, Japan).

Human postmortem tissue from AD patients and non-demented controls was obtained from the Radboud University Medical Centre (Department of Pathology, Nijmegen, The Netherlands, see Table 1). Brain tissues were fixed for 1 month after which the unembedded tissue was cut on a Vibratome in 50 μm thick sections. Sections were rinsed in TBS and subsequently treated with a graded series of methanol (20, 40, 60, 80 and 100 %, 10 min each), and back to 20 % methanol with 0.3 % H_2_O_2_ for 30 min and for 1 h in TBS. Sections were then incubated overnight at 4 °C with antibodies against misfolded Tau (MC1, 1:100, and UBB^+1^ (Ubi2A, 180398, 1:500). All dilutions were in 0.05 M Tris buffered saline (0.15 M with 0.25 M gelatin and 0.5 % Triton X-100, pH 7.4) [10, 41]. Subsequently, sections were incubated with donkey–anti-mouse or donkey–anti-rabbit antibodies (Jackson Laboratories), both biotinylated (1:400) and ABC (Vector, 1:400) and subsequently stained with DAB as described above.

**Table 1:** Clinico-pathological information of non-demented controls and AD patients

Number	Age (years)	Sex (F/M)	Braak stage^a^	Neurofibrillary tangles brainstem^b^		Amyloid^a^	Brain weight (g)
				LC	PBNl		
1	52	M	0	+	−	0	1,424
2	59	F	0	+	−	0	1,523
3	87	M	3	++	−	Sparse	1,245
4	68	M	5	+++	++	Frequent	1,312
5	63	M	6	+++	++	Frequent	980
6	64	F	6	+++	++	Frequent	1,250

All specimens were fixed in 4 % buffered formalin for 6 weeks

*LC* locus coeruleus, *PBNl* lateral parabrachial nucleus, − no immunoreactivity of neurofibrillary tangles or neuropil threads, + minor immunoreactivity of neurofibrillary tangles or neuropil threads, ++ considerable immunoreactivity of neurofibrillary tangles or neuropil threads, +++ intense immunoreactivity of neurofibrillary tangles or neuropil threads

^a^Based upon examination of temporal cortex and hippocampus

^b^Based upon MC1 staining for misfolded Tau

## Results

### Systemic phenotyping in the German Mouse Clinic

The primary phenotyping protocol in the German Mouse Clinic (GMC) included the measurement of more than 240 parameters in the areas of allergy, behavior, bone and cartilage, cardiovascular diseases, clinical chemistry, energy metabolism, eye development and vision, immunology, respiratory function, molecular phenotyping, neurology, nociception, and pathology [13, 15, 16]. As we expected that some mutant phenotypes might be associated with the aging process, UBB^+1^ tg mice and control animals were analyzed for phenotypes in two cohorts at the ages of about 3 and 12 months. Mice of both age cohorts were analyzed in the primary screening protocol within 8 weeks of the modal age. Significant phenotypic differences between UBB^+1^ tg and wild-type control mice that are presented here were found for respiratory function. In addition, we observed significantly altered parameters in metabolism, behavior and hippocampal gene expression, which are described in the supplementary information.

### Spontaneous breathing patterns

**Fig. 1:**
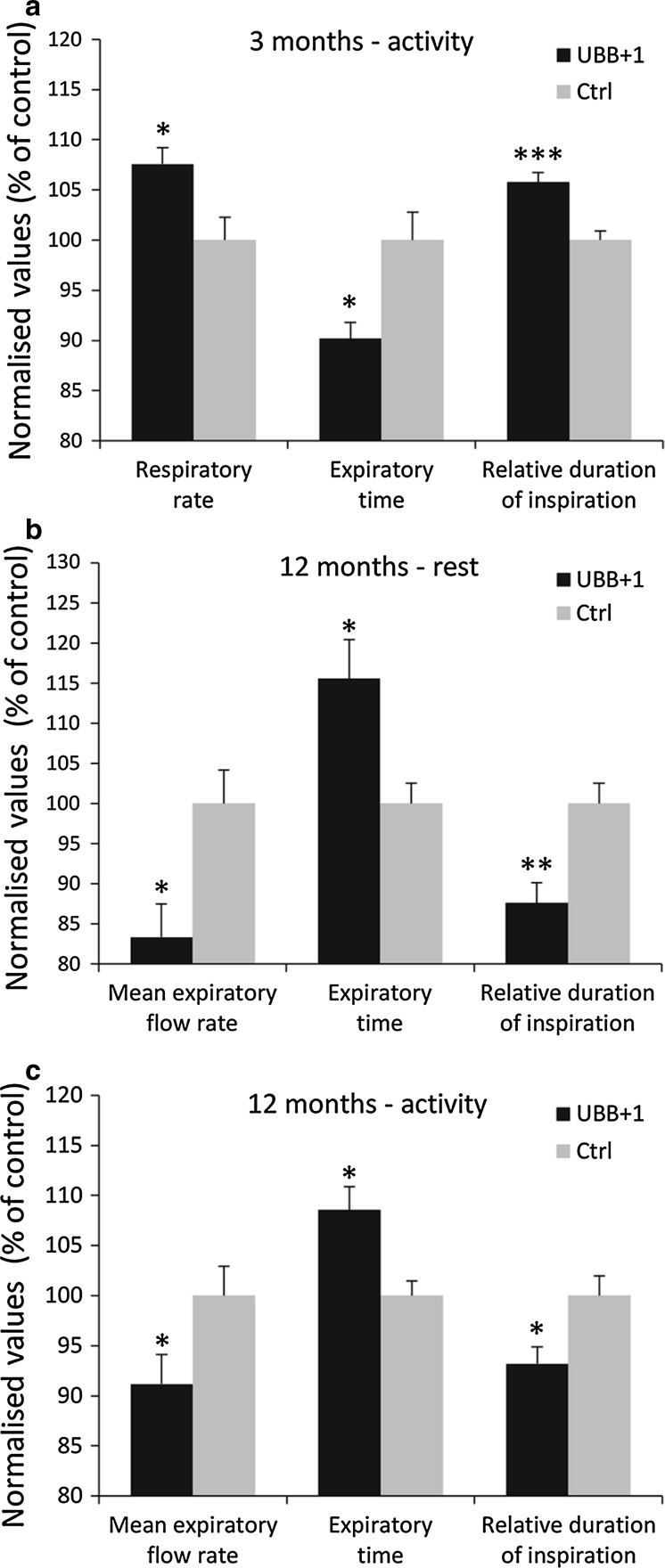
UBB^+1^ tg spontaneous breathing patterns measured by whole body plethysmography. Male UBB^+1^ tg (*black bars*) and control mice (*grey bars*) at the age of **a** 3 months and **b**, **c** 12 months were analyzed (*n* = 6 for all groups). **a** Shows the respiratory rate (f), expiratory time (Te), and relative duration of inspiration (Ti/TT) for male mice under activity. **b**, **c** Show the mean expiratory flow rate (MEF), expiratory time (Te), and the relative duration of inspiration (Ti/TT) at the age of 12 months at rest and under activity, respectively. The mean values for the indicated parameters of the control group were set to 100 % and significance by Student’s *t* test is indicated by *p<0.05, **p<0.01, ***p < 0.001; *error bars* represent SEM

Spontaneous breathing patterns were measured at rest and at activity, i.e., during calm sitting and short periods of sleeping and while animals were exploring the test chamber, respectively. Data were analyzed for each gender separately. At 3 months of age, significant genotype-dependent respiration differences between control mice and UBB^+1^ tg mice were detected for male mice during activity. In this group, the respiratory rate in UBB^+1^ tg mice was 8 % higher mostly due to a shorter expiratory time (Fig. 1a; S4). In addition, a slightly increased relative duration of inspiration was also observed in male UBB^+1^ tg mice compared to wild-type control mice (6 % increase, *p* < 0.001). The spontaneous breathing of UBB^+1^ tg mice was even more affected at the age of 12 months when respiratory timing was significantly altered in male UBB^+1^ tg mice during activity and at rest (Fig. 1b, c). The trend towards longer time of expiration and shorter relative duration of inspiration during aging was consistent among UBB^+1^ tg males (Fig. 1b, c; Fig. S5) and females (Fig. S1, S5) in comparison to the respective wild-type control groups. At 12 months of age, male UBB^+1^ tg mice exhibited significantly shorter relative duration of inspiration at rest (90 ± 3 %, Fig. 1b) and during activity (94 ± 3 %, Fig. 1c) compared to male wild-type control mice (set to 100 %). Since the inspiratory time and the mean inspiratory flow rate were not significantly affected in male UBB^+1^ tg mice (Fig. S5), the observed shorter relative duration of inspiration values was primarily due to an elevated expiratory time. The mean expiratory flow rate was significantly lower at rest (83 ± 4 %) and activity (91 ± 4 %) in UBB^+1^ expressing tg mice compared to the corresponding male control group (set to 100 %), which is most likely due to the differences in the expiratory time (Fig. 1b, c). Thus, overall the spontaneous breathing pattern of male transgenic UBB^+1^ mice was affected by a statistically significant increase of the expiratory time over age. In contrast, the male wild-type control group had a tendency towards reduced expiratory time over age. Female transgenic mice at 12 months of age and male transgenic mice at 18 months of age showed similar trends, which became significant for the latter (*p* < 0.05; Fig. S1, S2). Absolute values for the spontaneous breathing parameters are provided in the supplementary figures S4, S5 and S6.

The altered spontaneous breathing pattern of the UBB^+1^ transgenic mice is not a mutant phenotype that causes obvious or severe suffering of the mice. The UBB^+1^ transgenic mice appear externally healthy and it is a mutant phenotype that is not apparent by gross inspection of the mice. However, within the primary lung screen of the German Mouse Clinic, which assesses the spontaneous breathing pattern by whole body plethysmography, we rarely observe such a consistent and clear mutant phenotype at least among mouse lines that externally appear rather normal.

To exclude the possibility that the observed differences in breathing patterns between transgenic and wild-type mice were merely caused by altered lung function per se, we tested various lung function parameters, including lung volume, mechanics, and gas exchange. None of the measured parameters were altered in UBB^+1^ tg mice compared to the wild-type control mice (Fig. S3), suggesting that differences in breathing pattern are not due to lung dysfunction per se but rather due to changes in respiratory regulation.

### Breathing response to hypoxic and hypercapnic conditions

The breathing pattern of vertebrates with lung respiration is tightly controlled by the partial pressures of carbon dioxide and oxygen in the blood. Peripheral and central chemoreceptors ensure that increased concentrations of blood carbon dioxide (hypercapnia) or decreased levels of blood oxygen (hypoxia) result in increased respiratory efforts. To test the functionality of these feedback loops and to further examine the relevance of the observed changes in the spontaneous breathing pattern, 18-month-old UBB^+1^ tg and control mice were exposed to hypercapnic or hypoxic conditions.

**Fig. 2:**
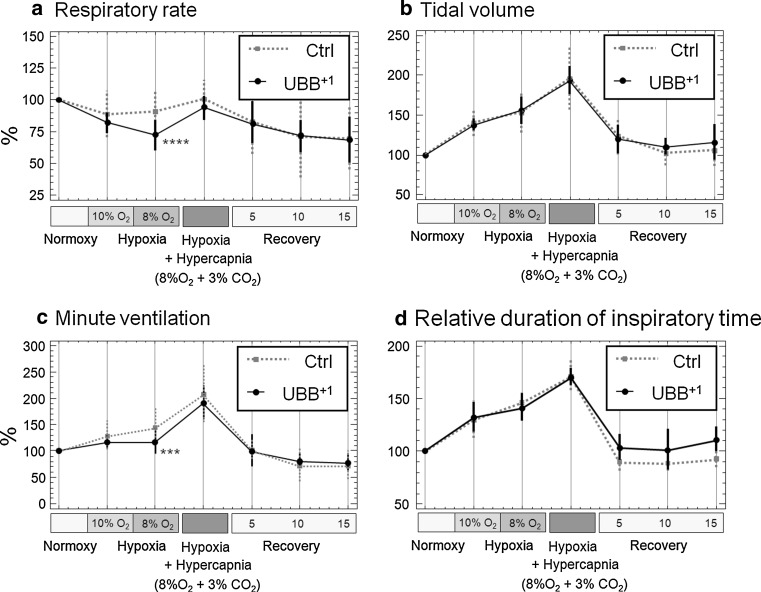
Ventilatory responses to hypoxic and hypercapnic conditions are affected by UBB^+1^ expression. Male mice at the age of 18 months (*n* = 8 for each group) were exposed to 10 and 8 % O_2_ (hypoxia) followed by a combined exposure of 8 % O_2_+3 % CO_2_ (hypoxia and hypercapnia) each for 7 min and a recovery period of 15 min. **a** Shows the respiratory rates (f), **b** shows the tidal volume (TV), **c** shows the minute ventilation (MV), and **d** shows the relative duration of inspiration time (Ti/TT). Values were calculated relative to baseline levels (set to 100 %) and significance by Student’s *t* test is indicated by ****p<0.001, ***p<0.01; *error bars* represent SEM. The response to hypercapnic conditions is shown in Supplementary Figure S7

Under hypoxic conditions, the most prominent response in all mice of the transgenic as well as the control group was observed for the tidal volume (Fig. 2b), which increased about 1.5-fold with 8 % O_2_ in the respiratory air in comparison to normal air (about 21 % O_2_). When hypoxic conditions were combined with hypercapnic conditions (3 % CO_2_ in the respiratory air), the tidal volume further increased almost twofold. The combined challenge of hypercapnic and hypoxic conditions also increased the relative duration of inspiration by about 170 % (Fig. 2d), indicating that it took the animals more time to inhale the increased tidal volume. All values returned to baseline conditions within a 15-min recovery period. Even though UBB^+1^ tg animals and control mice showed a similar increase in tidal volume under hypoxic conditions at 8 % O_2_, they showed significant differences in two other parameters under this condition. Thus, the respiratory rate in UBB^+1^ tg mice declined to about 75 % at 8 % O_2_ (206.7 ± 8.3 min^−1^) whereas this parameter was only slightly changed in control animals (about 90 %; 246.8 ± 8.4 min^−1^; *p* < 0.001; Fig. 2a). Secondary to this, the minute ventilation was also significantly lower in UBB^+1^ tg mice (Fig. 2c; *p* < 0.01). Interestingly, when adding the hypercapnic challenge of 3 % CO_2_ in the breathing air to the hypoxic challenge, the breathing patterns of UBB^+1^ tg and controls were not significantly different (Fig. 2a, c). In addition, we examined breathing patterns of mice exposed to 3, 5 and 8 % CO_2_ in the air (at normal oxygen conditions). Under these conditions, we did not observe differences between UBB^+1^ tg animals and wild-type mice (Fig. S7). These findings support the notion that the hypoxic response is primarily affected in UBB^+1^ transgenic mice. In contrast, the feedback loop for CO_2_ levels is apparently not affected in UBB^+1^ transgenic mice.

### Anatomic localization of UBB^+1^ in the mouse brain

**Fig. 3:**
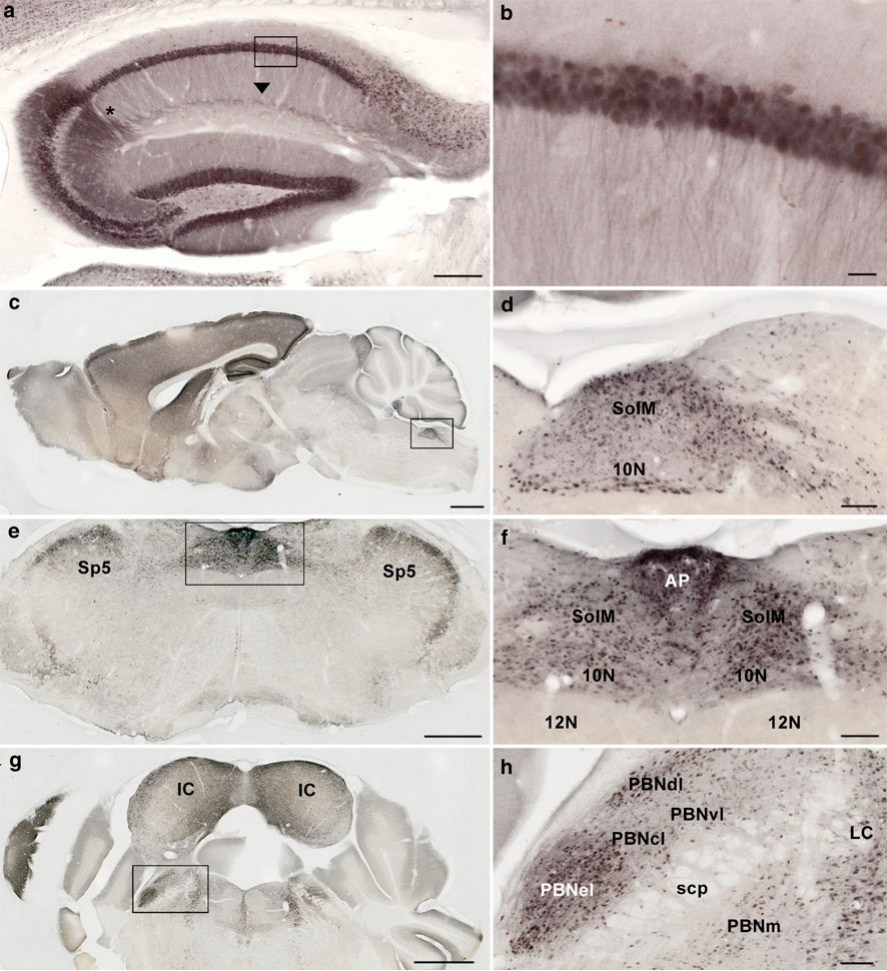
Immunohistochemical expression of UBB^+1^ in the brain of tg mice. UBB^+1^ staining of **a**, **b** hippocampus and **c**–**h** brainstem of male UBB^+1^ tg mice at the age of 15 months (hippocampus) and 3 months (brainstem) with the Ubi3 antibody (sagittal section, **a**/**b**). Hippocampal cells are immunoreactive for UBB^+1^. The fibers widespread in the hippocampus are also stained (see *boxed area* in **a**), especially in the region where the CA3 borders the CA2 (*asterisk*). Another region of more intense UBB^+1^ staining reflects the stratum lacunosum (*arrowhead*), apparently with crossing fibers of the perforant pathway. Sagittal (**c**, **d**) and coronal (**e**–**h**) sections of UBB^+1^ tg mice were incubated with Ubi3. **c** Note the expression of UBB^+1^ in the dorsal vagal complex (nucleus tractus of the solitarius, area postrema, dorsal motor nucleus of vagus nerve). **d** Region of dorsal vagal complex as indicated by the *boxed area* in **c** at a higher magnification. **e** Coronal section shows again the expression of UBB^+1^ in the dorsal vagal complex and **f** shows this region (*boxed area* in **e**) at a higher magnification. Note in **e** UBB^+1^ immunostaining in the spinal 5 nucleus. **g** Note the expression of UBB^+1^ in neurons of the different subnuclei of the parabrachial nucleus (*boxed area*). **h** Photograph of parabrachial nucleus at a higher magnification. Nomenclature based upon [14]. Abbreviations see paper. *Scales*: **a** = 200 μm, **b** = 20 μm, **c** = 1 mm, **d** = 100 μm, **e** = 500 μm, **f** = 100 μm, **g** = 1 mm, **h** = 100 μm

The expression of UBB^+1^ in brain sections of UBB^+1^ tg mice was examined using the UBB^+1^-specific Ubi3 antibody (bleeding 16-9-97) and a more sensitive procedure (avidin–biotin method) than previously used [11]. In the present study, hippocampal fibers in the CA region were positive, especially in the CA2 region (Fig. 3a, b). The intense fiber staining in the region where the CA3 borders the CA2 appears to reflect an increased fiber density. The incubation of mouse brain tissue with ^6^His tag UBB^+1^ and the pre-immune control serum resulted in an absence of immunopositive staining (data not shown). There was no staining in the brains of wild-type control mice.

The present immunohistochemical staining procedure also enabled a superior anatomic localization of UBB^+1^ in other brain regions as compared to our previously published results [11]. In particular, in the brainstem of UBB^+1^ tg mice labeling with the Ubi3 antibody was prominent in regions associated with known functions in respiratory modulation (Fig. 3c–h). Both in the dorsal respiratory group (Fig. 3c–f) and the pneumotaxic center (Fig. 3g, h), UBB^+1^ immunoreactive nuclei were found. In UBB^+1^ tg animals, UBB^+1^ was present in specific areas of the dorsal vagal complex, such as the area postrema (AP), the nucleus of the tractus solitarius (NTS), and the dorsal motor nucleus of the vagus nerve (Fig. 3c–f). Strongly positive UBB^+1^ immunoreactive neurons were also present in the locus coeruleus (LC) and subnuclei of the parabrachial nucleus (PBN) (Fig. 3g, h), especially in the external lateral (PBNel) and dorsal lateral (PBNdl) subnuclei, which receive visceral inputs from the caudal NTS. The rodent external lateral nucleus is involved in respiratory function and in the modulation of respiratory responses to hypoxia and hypercapnia [35, 36]. In particular, the central respiratory control of the response to hypoxia was affected in UBB^+1^ tg mice (Fig. 2). These findings are consistent with the hypothesis that highly elevated UBB^+1^ expression might have mild toxic effects that could interfere with the normal neuronal function [42]. Mice were analyzed for UBB^+1^ expression at the ages of 3, 6, 15 and 18 months, but no differences among the four ages were found with respect to UBB^+1^ immunohistochemical staining, which is in agreement with previously published data [11].

### Anatomic localization of UBB^+1^ in the human brain

**Fig. 4:**
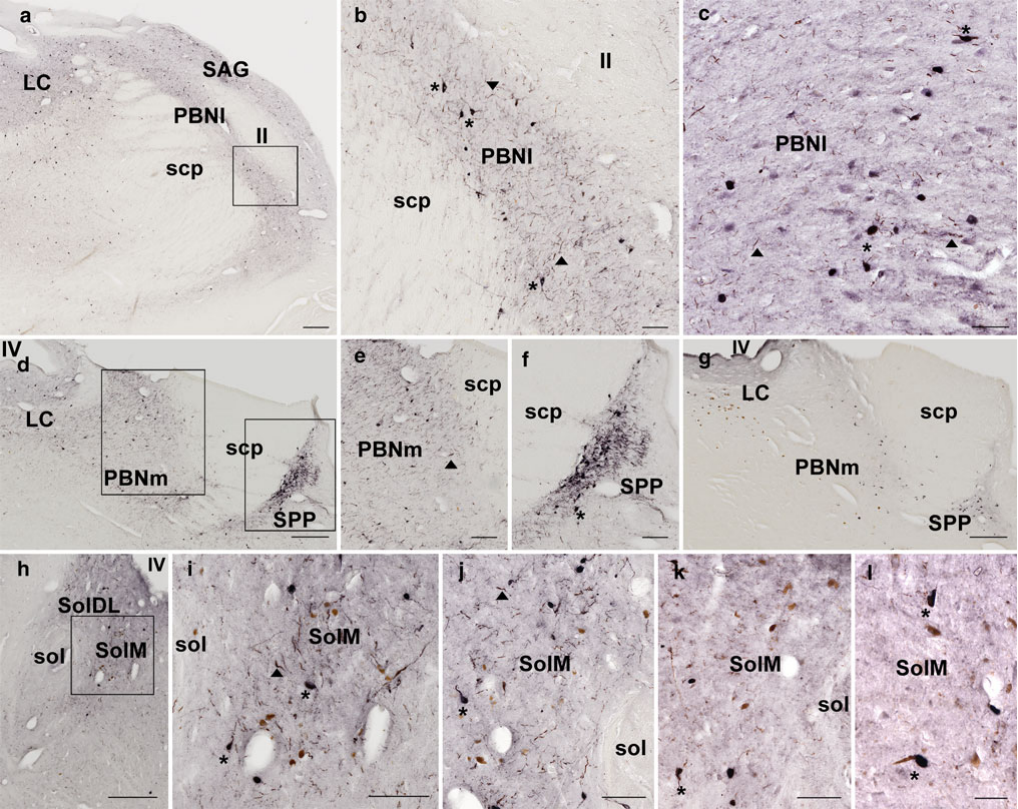
Immunohistochemical expression for misfolded Tau and UBB^+1^ in the human brainstem. Intense staining (MC1) for misfolded Tau (**a**, **c**–**f**, **h**–**j**) and UBB^+1^ (**c**, **g**, **k**, **l**) in various nuclei of the brainstem of patient # 5 (Braak stage 6). **a**–**c** Oral pons (Obex ± 25 mm, [32]) and **d**–**g** Oral pons (Obex ± 30 mm). **b**, **e** and **f** are larger magnifications of **a** and **d** respectively, as indicated by the *boxes* in **a** and **d**. Neurofibrillary tangles (*asterisk*) and neuropil threads (*filled triangle*) are stained in the locus coeruleus (LC), the medial (PBNm) and lateral parabrachial nucleus (PBNl). The PBNl is located between the superior cerebellar peduncle (scp) and the lateral lemniniscus (ll). The PBNm and subpeduncular pigmented nucleus (SPP) are located, medially and ventrolaterally respectively, to the scp. **h**–**l** More caudally in the medulla (Obex ± 5 mm) the ventral (SolM), dorsolateral (SolDL), other solitary subnuclei and the dorsal motor nucleus of the vagus show neurofibrillary tangles (**h**–**j**), that are UBB^+1^ positive (**k**, **l**). **i** is a larger magnification of the *boxed area* in (**h**). Note *brown* neuromelanin inclusions in **h**–**l**. *SAG* sagulum, *sol* solitary tract. *Scales*: **a**, **d**, **g**, **h** = 500 μm; **e**, **f**, **i**–**k** = 200 μm; **b**, **c** = 100 μm; **l** = 30 μm. *Asterisk*, neurofibrillary tangles by MC1 staining; *filled triangle*, neuropil threads

In the brainstem of sporadic AD patients (Braak stage #6 and less prominent in stages #3 and #5) comparable nuclei as in the UBB^+1^ tg mouse line 3413 mouse were stained positively for misfolded Tau and UBB^+1^ in the pneumotaxic centers of the brain (Fig. 4a–g), e.g., in the locus coeruleus, subcoeruleus, the lateral and medial parabrachial nuclei, the subpeduncular pigmented nucleus and the sagulum. Many neurofibrillary tangles, kinky and curly fibers can be seen (Fig. 4b, f, g). In the dorsal respiratory group, UBB^+1^ immunoreactivity was found in the nuclei surrounding the solitary tract (Fig. 4h, i). These stainings were not observed in stages #0. Also in the medulla, various nuclei around the solitary tract were immunoreactive for misfolded Tau (Fig. 4h–j) and UBB^+1^ (Fig. 4k, l). These anatomical data support the possibility of respiratory dysfunction in AD.

## Discussion

The precise role of the UPS in neurodegeneration and aging is not clear, but the partial impairment of its function in the mouse brain results in spatial memory deficits and changes in fear-related behavior [11, 24, 28]. In the present study, we extended these data using additional behavioral tests on UBB^+1^ tg mice (see also supplementary data) and report further phenotypic differences. Moreover, the intensity of the UBB^+1^ staining was remarkably enhanced by the improved immunohistochemical staining protocol enabling better localizations (Fig. 3a, b). Our data suggest that neurons involved in the central control of respiration are sensitive to long-term UPS inhibition via the expression of the UBB^+1^ protein. Although the observed differences were significant, the postnatal transgenic expression of UBB^+1^ seems to be rather well tolerated by mice, because the vast majority of the more than 240 parameters measured in the primary phenotyping protocol of the German Mouse Clinic did not show significant differences in comparison to control animals. This limited transgenic phenotype is in accordance with previous studies [11] where no signs of protein aggregations, neurodegeneration or other histological changes were observed. This finding might be explained by the rather modest reduction of proteasomal activity (at least 20 %) in brain tissue of UBB^+1^ tg mice [11]. The UBB^+1^ tg mouse line is to our knowledge the only existing in vivo model for the long-term, low level inhibition of the UPS. This feature models the human situation during aging and neurodegeneration, where chronic reduction of the UPS activity is observed [25]. To approximate the situation in humans, our systematic phenotyping was done with UBB^+1^ tg mice at 3 and 12 months of age. In both age groups, the same biological functions, namely respiratory function, behavior and metabolism were affected in the UBB^+1^ transgenic mice. In contrast to humans, a dramatic increase in the severity of the transgenic phenotype with age was not observed, which is in line with previous results [11] and which may be related to the relatively short life span of mice.

### UBB^+1^ phenotype affects respiratory function

The key findings of the respiratory function analysis were related to altered breathing patterns particularly in male UBB^+1^ tg mice, which affected the respiratory rate and respiratory timing. Respiratory timing was predominantly affected by a reduction of the relative duration of inspiration (Ti/TT) in combination with an increased expiratory time (Te). An affected breathing pattern per se is a rather unspecific finding, which could be due to several factors, (1) altered central breathing control, (2) impaired sensitivity of peripheral chemoreceptors, (3) altered respiratory muscle function, or (4) affected respiratory function per se. To address these different issues, we tested UBB^+1^ tg mice for respiratory lung function (lung volumes, mechanics, and gas exchange) and the ventilatory response to hypercapnic and hypoxic conditions, respectively. With regard to lung function, the parameters were not different in UBB^+1^ tg and wild-type mice. These findings indicated that the increased expiratory time is not related to an increased airway resistance that the gas exchange function of the respiratory system per se (4) is not affected by UBB^+1^ transgenic overexpression and that the alterations in breathing pattern are not caused by impaired respiratory muscle function (3). This conclusion is further supported by the hypercapnic and hypoxic challenge responses. A similar increase up to a factor of 2 in tidal volume in controls and UBB^+1^ tg mice was observed indicating that UBB^+1^ tg animals have normal thoracic muscle strength. To further distinguish between central (1) and peripheral (2) chemoreceptor sensitivity, mice were exposed to hypercapnic and/or hypoxic conditions and the ventilatory response was measured. The results show that the hypercapnic response was not affected in UBB^+1^ tg mice, while the hypoxic response was. UBB^+1^ tg mice showed declining respiratory rates with increasing levels of hypoxia, being 16.3 % lower than in controls at 8 % O_2_. As a result, minute ventilation was also significantly lower in UBB^+1^ tg mice. Interestingly, breathing differences between UBB^+1^ tg and control mice were virtually absent when the hypercapnic challenge of 3 % CO_2_ was added to the hypoxic challenge. This supports the conclusion that primarily the hypoxic feedback loop is affected in UBB^+1^ tg mice. Hence, these experimental results suggest that an altered central breathing control (1) is the most likely explanation for the observed phenotype.

In support of this hypothesis, we observed strong UBB^+1^ tg expression in various brainstem nuclei which are involved in the regulation of respiratory function. For example, the nucleus of the solitary tract (NTS), which is part of the dorsal respiratory group, was UBB^+1^ immunoreactive in all the different subnuclei. The caudal part of this nucleus receives afferent information from baroreceptors, mechanoreceptors and from central and peripheral chemoreceptors. Furthermore, it has been shown that the AP together with NTS project to the PBN [20], which comprises a second respiratory center positive for UBB^+1^ expression. Specifically, the external lateral and dorsal lateral PBN subnuclei were strongly UBB^+1^ immunoreactive. It has been reported that the external lateral PBN subnucleus in rodents has an important role in the respiratory response to hypoxic and hypercapnic conditions [35, 36]. This is of particular interest because UBB^+1^ tg mice showed differences in their response to hypoxia compared to the controls. Interestingly, it has previously been shown that the locus coeruleus area and the PBN are among the early targets of the AD-related cytoskeletal pathology, which suggests that parts of the brainstem respiratory centers are affected during AD progression [4, 33]. UBB^+1^ tg mice are not the only mouse model with altered respiration. For example, *Tau*-P301L transgenic mice show a breathing phenotype, which can be linked to *Tau* (*Mapt*) expression in defined nuclei of the brainstem such as the Kölliker-Fuse nucleus [8, 29]. Apart from respiratory dysfunction, swallowing impairments are common in AD patients and frequently lead to aspiration pneumonia [23]. The three phases of swallowing (oral, pharyngeal and esophageal) are regulated by central pattern generators in the brainstem and one of them is the NTS [27]. This suggests that there might be a functional link between the respiratory changes exhibited by the UBB^+1^ tg mice and the respiratory and swallowing dysfunctions in AD patients, because similar anatomical regions are affected as we have shown in our examination of the distribution of UBB^+1^ in the brains of AD patients. Intriguingly, it was also recently shown that the minute volume in AD patients under peak exercise conditions is reduced in comparison to a non-demented control group [3]. Our data suggest that this change in respiratory response in AD patients could also be due to a dysfunction of central breathing control.

### Conclusions

Our comprehensive phenotyping approach revealed a respiratory phenotype in UBB^+1^ tg mice, which prompted us to perform an extensive immunohistochemical UBB^+1^ analysis of the brainstem of these mice. In several areas involved in respiratory regulation such as the lateral parabrachial nuclei [35, 36] and the dorsal vagal complex, we detected intense UBB^+1^ immunoreactivity. Our data suggest that also in other AD mouse models respiratory function analysis needs to be addressed.

As UBB^+1^ tg mice are an animal model for early AD, we subsequently analyzed UBB^+1^ expression in AD patients and healthy individuals. In the human brainstem, UBB^+1^ immunoreactivity and misfolded Tau were seen in similar areas as in UBB^+1^ tg mice and they may well contribute to the selective vulnerability of the brainstem to AD [31]. A role of the lateral parabrachial nucleus in mediating the feedback control of inspiratory drive has been reported [35]. The dorsal respiratory group communicates with this area and shows UBB^+1^ immunoreactivity as well. It could well be that breathing dysfunction may play a major role in cognitive, behavioral and emotional impairments in early onset AD [22]. Also, a disproportional contribution of bronchopneumonia as a cause of death in AD patients is consistent with our findings that indicate breathing dysfunction as a contributing factor. With respect to respiratory function in aged patients and during AD per se, it has been noted that increased cardiorespiratory fitness in early-stage AD is associated with reduced brain atrophy as compared with non-demented individuals [6]. Moreover, it was recently reported that aerobic exercise has positive effects on mild cognitive impairment (e.g., [1]), underscoring the need to enhance respiratory function. Together with these published observations, our data might suggest that the examination of the respiratory system could be an important parameter for monitoring the progression of AD. It remains to be analyzed whether other cognitive disorders also display UBB^+1^ expression and neuronal pathology in central breathing control regions.

## Electronic supplementary material

Below is the link to the electronic supplementary material.

Supplementary material 1 (DOC 1950 kb)

## Abbreviations

Ub: Ubiquitin
UBB: Ubiquitin B
UBB^+1^: Mutant ubiquitin B^+1^
UPS: Ubiquitin–proteasome system
AD: Alzheimer’s disease
PD: Parkinson’s disease
APP: Amyloid precursor protein
Ti/TT: Relative duration of inspiration
AP: Area postrema
IC: Inferior colliculus
LC: Locus coeruleus
ll: Lateral lemniscus
NTS: Nucleus of the tractus solitarius
PBN: Nucleus parabrachialis
PBNcl: Nucleus parabrachialis central lateral
PBNdl: Nucleus parabrachialis dorsolateral
PBNel: Nucleus parabrachialis external lateral
PBNem: Nucleus parabrachialis external medial
PBNl: Lateral parabrachial nucleus
PBNm: Medial parabrachial nucleus
PBNvl: Nucleus parabrachialis ventrolateral
scp: Superior cerebellar peduncle
sol: Solitary tract
SAG: Sagulum
SolDL: Nucleus of the tractus solitarius, dorsolateral part
SolM: Nucleus of the tractus solitarius, medial part
SPP: Subpeduncular pigmented nucleus
Sp5: Spinal 5 nucleus
10N: Dorsal motor nucleus of the vagus nerve
12N: Hypoglossal nucleus
IV: Fourth ventricle
