# Supplementary material for: Long-term proteasomal inhibition in transgenic mice by UBB+1 expression results in dysfunction of central respiration control reminiscent of brainstem neuropathology in Alzheimer patients
Source: Acta Neuropathol. 2012 Jun 23;124(2):187–97. doi: 10.1007/s00401-012-1003-7 (PMC3400757; doi:10.1007/s00401-012-1003-7)
Supplement: Supplementary file 1 — Supplementary material 1 (DOC 1950 kb) [file 401_2012_1003_MOESM1_ESM.doc]

Long-term proteasomal inhibition in transgenic mice
by UBB^+1^ expression results in
dysfunction of central respiration control reminiscent of
brainstem neuropathology in Alzheimer patients

**SUPPLEMENTS**

- **Supplementary methods**
  - Analysis of lung function
  - Behavioral analysis
  - Metabolic phenotyping
  - RNA isolation and transcriptome analysis of the whole hippocampus
  - Immunoblot analysis of synaptic markers from hippocampus tissue
  - Microarray analysis of the CA3 region
- **Supplementary results**
  - Behavioral changes
  - Metabolic changes
  - Gene expression changes in the hippocampal formation
  - Transcriptomic changes in the hippocampal CA3 region
- **Supplementary discussion**
  - Behavioral changes and hippocampal gene expression
  - Metabolic changes
  - Transcriptomic and proteomic changes in the hippocampus
- **Supplementary figures**
  - Fig. S1. Spontaneous breathing pattern in female mice at the age of 12 months
  - Fig. S2. Spontaneous breathing pattern in male mice at the age of 18 months
  - Fig. S3. Lung function is not altered in male UBB^+1^ tg mice at the age of 18 months
  - Fig. S4. Data for spontaneous breathing pattern at the age of 3 months
  - Fig. S5. Data for spontaneous breathing pattern at the age of 12 months
  - Fig. S6. Data for spontaneous breathing pattern at the age of 18 months
  - Fig. S7. Ventilatory response to hypercapnic conditions at the age of 18 months
  - Fig. S8. UBB^+1^ tg mice behave differently than wt mice
  - Fig. S9. Lower food intake in female UBB^+1^ tg mice at 3 months of age
  - Fig. S10. Less metabolisable energy in UBB^+1^ tg mice at 3 months of age
  - Fig. S11. Diminished weight loss in UBB^+1^ tg mice upon a fasting challenge
  - Fig. S12. Regulation of synaptic markers in the hippocampus
  - Fig. S13. Genes not significantly regulated in UBB^+1^ tg mice versus controls
- **Supplementary tables**
  - Table S1. 274 genes are significantly (FDR<10%) regulated in the CA3 region of UBB^+1^ tg mice compared to control animals
  - Table S2. Selected significantly enriched functional annotations associated with the 274 genes regulated in the CA3 region of UBB^+1^ tg mice
  - Table S3. UPS-related genes regulated in the CA3 region of UBB^+1^ tg mice: biological functions and associated diseases
  - Table S4. Genes tested for mRNA expression by qPCR
  - Table S5. Sequences of the oligos used for qPCR
- **Supplementary abbreviations**
- **Supplementary references**

**Supplementary methods**

**1. Analysis of lung function**

Lung function measurements are described in detail elsewhere and are briefly introduced here.

**1.1 Whole body plethysmography**

A commercially available system from Buxco® Electronics (Sharon, Connecticut) was used to assess breathing patterns in unrestrained animals according to the principle described by Drorbaugh and Fenn (1955). It measures the pressure changes, which arise from inspiratory and expiratory temperature and humidity fluctuations during breathing. Calibration of the system allows to transform these pressure swings into flow and volume signals so that automated data analysis provides tidal volumes (TV), respiratory rates (f), minute ventilation (MV), inspiratory and expiratory times (Ti, Te), as well as peak inspiratory and peak expiratory flow rates (PIF, PEF). These data were stored online as mean values at 10 s intervals. Measurements were always performed between 8 a.m. and 11 a.m. to account for potential diurnal variations in breathing. The system was set up in a quiet room where temperature and humidity were kept constant throughout the measurements. Before each measurement, the system was calibrated and the actual barometric pressure, temperature, and humidity were supplied to warrant adequate calculations of flow rates and volumes. After placing the animals into the chamber, data recording was immediately started and was continued for 40 min. Mice underwent typical phases during the measuring period. Primarily, the animals were stressed so that the respiratory rate was highest at the beginning. Usually after 5 min. the animals became calmer, they slightly reduced their respiratory rate, and began to explore the chamber and start cleaning themselves – *phase of activity*. Later activity was more and more interrupted by phases of rest or even short periods of snoozing – *resting phase.* The frequency histogram of the respiratory rates was determined for each individual, and breathing was analyzed for the above mentioned parameters during the phases of activity and rest. In addition to the directly recorded parameters, mean inspiratory and expiratory flow rates (MIF, MEF) were calculated offline from the ratio of tidal volume and the respective time interval. The relative duration of inspiration (Ti/TT) was determined from the ratio of inspiratory time to total time required for the breathing cycle. Specific tidal volumes and minute ventilations (sTV, sMV) were calculated by relating the absolute values to the body weight of the animal.

**1.2 Lung function**

The anesthetized and intubated mouse was connected to a custom made computer-controlled piston-type servo ventilator which provides positive pressure ventilation and measures of lung volume and its subdivisions, respiratory mechanics (compliance and resistance), intrapulmonary gas mixing and alveolar-capillary gas transfer. Miniaturized pressure transducers were used to continuously measure airway opening pressure and oesophageal pressure. Concentrations of oxygen, carbon dioxide, labeled carbon monoxide (C^18^O), and helium are measured by a magnetic sector field mass spectrometer (modified M3, Varian MAT). During lung function measurements, the signals of interest were digitized and stored in a personal computer at a rate of 100-500 Hz. Before data analysis, the output signals of the mass spectrometer were corrected for the lag times of the mass spectrometer to obtain real time data. All measurements were performed in duplicate and the mean of both was used for further calculations. Lung function measurements include:

(i) *Lung volumes:* TLC (µl) total lung capacity; TLC/bw (µl/g) specific total lung capacity; IRC (µl) inspiratory capacity; FRC (µl) functional residual capacity; FRC/TLC relaxation volume; ERV (µl) expiratory reserve volume; VD (µl) conducting airway volume, i.e. series (Fowler) dead space volume from helium exspirogram; VD/TLC specific airway volume

(ii) *Respiratory mechanics*: Compliance and resistance: Cdyn (µl/cmH2O) dynamic compliance of respiratory system; CLdyn (µl/cmH_2_O) dynamic lung compliance; C (µl/cmH2O) static compliance of respiratory system; CL/TLC specific compliance of the respiratory system (µl/cmH_2_O/ml TLC); CL (µl/cmH2O) static lung compliance; CL/TLC specific lung compliance (µl/cmH2O/ml TLC); R (cmH_2_O/ml/s) respiratory system resistance; sR (cmH2O/s) specific respiratory system resistance (R x TLC)

(iii) *Intrapulmonary gas mixing*: SHe (mmHg/ml) slope of the alveolar plateau (phase III) from helium expirogram

(iv) *Alveolar-capillary gas transfer:* DCO (µmol/min/hPa) diffusing capacity for carbon monoxide; DCO/TLC specific diffusing capacity (mol/min/hPa/ml TLC).

**1.3 Ventilatory response to hypoxia and hypercapnia**

Assessing the ventilatory response to hypoxia and hypercapnia is a well-established method to detect disturbances in the regulation of respiration in man and in rodents . The commercially available system from Buxco® Electronics (Sharon, Connecticut) was modified and used to assess changes in breathing pattern in unrestrained animals while being challenged with hypercapnia or/and hypoxia. Following a standardized protocol animals were exposed to different levels of CO_2_ (3%, 5%, 8% CO_2_) for 7 minutes at each level. After a recovery period of 15 minutes mice were exposed to different levels of hypoxia (10% or 8% O_2_) or a combination of hypoxia and hypercapnia (8% O_2_ and 3% CO_2_), once more for 7 minutes at each level of exposure. The ventilatory response was assessed from the breathing pattern determined at each level of hypercapnic or hypoxic exposure during the 6^th^ and 7^th^ minute where stable breathing conditions had been reached. The following parameters were determined: Tidal volumes (TV), respiratory rates (f), minute ventilation (MV), inspiratory and expiratory times (Ti, Te), as well as peak inspiratory and peak expiratory flow rates (PIF, PEF), mean inspiratory flow rates (MIF), expiratory flow rates (MEF), relative duration of inspiration (Ti/TT), specific tidal volumes (sTV), minute ventilations (sMV).

**1.4 Statistical analysis of data**

Statistical analyses were performed using a commercially available statistics package (Statgraphics^®^, Statistical Graphics Corporation, Rockville, MD). Differences between strains were evaluated by Students t-test. Statistical significance was assumed at p<0.05. Data are presented as mean values ± standard error of the mean (SEM).

**2. Behavioral analysis**

**2.1 Behavioral phenotyping**

For the modified Hole Board test (see detailed descriptions below and ) the animals were separated based on sex, but not genotype. Mice were analyzed at the age of 8-10 weeks (males: 15 controls and 15 transgenics; females: 15 controls and 14 transgenics) and at the age of 47-52 weeks (females: 15 controls, 15 transgenics; males: 15 controls, 14 transgenics). Mice were housed for two weeks in the German Mouse Clinic for acclimatization before testing. Three days before testing, an object (metal cube) was placed into the home cage and removed one day before testing.

Spontaneous alternation performance was tested at the age of 6 months (males: 14 controls and 13 transgenics) by using a symmetrical Y-maze. Each mouse was placed in the center of the Y-maze and was allowed to explore the maze freely during a 6-min session. The sequence and total number of arms entered were recorded. Percentage alternation is the number of triads containing entries into all three arms divided by the maximum possible alternations (the total number of arms entered minus 2) X 100. Data were statistically analyzed by analysis of variance (ANOVA) using SPSS software (SPSS Inc., Chicago, USA). For all analyses the chosen level of significance was p<0.05 and data are presented as mean values ± standard error of the mean (SEM). For the measurement of activity parameters females and males were grouped if there was no sex x genotype interaction, and not grouped, where there was one (i.e. in object exploration parameters).

**2.2 Modified Hole Board**

The modified Hole Board test allows the comprehensive analysis of a range of parameters known to be indicative of behavioral dimensions such as locomotor activity, exploratory behavior, arousal, emotionality, memory and social affinity in a single short test . It was carried out as previously described . The test apparatus consisted of a test arena (100 x 50 cm), in the middle of which a board (60 x 20 x 2 cm) with 23 holes (1.5 x 0.5 cm) staggered in three lines with all holes covered by movable lids was placed, thus representing the central area of the test arena as an open field. The area around the board was divided into 12 similarly sized quadrants by lines taped onto the floor of the box . Both box and board were made of dark grey PVC. All lids were closed before the start of a trial. For each trial, an unfamiliar object (a blue plastic tube lid, similar in size to the metal cube) and the familiar object (metal cube) were placed into the test arena with a distance of 2 cm between them. The familiar objects had been placed into the animals’ home cages 3 days prior to testing, and were removed one day before testing. The illumination levels were set at approximately 150 lux in the corners and 200 lux in the middle of the test arena.For testing, each animal was placed individually into the test arena and allowed to explore it freely for 5 min. The animals were always placed into the test arena in the same corner next to the partition, facing the board diagonally. The two objects were placed in the corner quadrant diametrical to the starting point. Exploration of an object was defined as sniffing or biting it, or touching it with the forepaws. During the 5 min trial, the animal’s behavior was recorded by a trained observer with a hand-held computed. Data were analyzed by using the Observer 4.1 Software (Noldus, Wageningen). Additionally, a camera was mounted 1.20 m above the center of the test arena, and the animal’s track was videotaped and its locomotor path analyzed with a video-tracking system (Ethovision 2.3, Noldus, Wageningen). After each trial, the test arena was cleaned carefully with a disinfectant.

**3. Metabolic phenotyping**

Mice were analyzed at a mean age of 18 weeks (males: 7 controls, 7 transgenics; females: 6 controls, 7 transgenics) and at a mean age of 60 weeks (males: 7 controls, 7 transgenics; females: 7 controls, 7 transgenics). During the test, all mice were single caged on plastic grid panels (0.5 cm grid hole diameter) to allow the collection of feces and spilled food. They were fed *ad libitum* for a period of 14 days. Mice 60 weeks of age were subsequently challenged by two days of food deprivation. Water was available *ad libitum* at all times. During the different feeding regimes body weight, food consumption (F_con_), rectal temperature (T_re_), daily feces production (Fec), and the energy content of the feces (E_fec_) were measured, whereas energy uptake (E_up_), metabolizable energy (E_met_) and the food assimilation coefficient (F_ass_) were calculated from raw data. For the bomb calorimetric analysis, all egested feces was collected and separated from spilled food in three day intervals. Samples of lab chow and feces (~1 g) were dried at 60°C for two days, homogenized in a coffee grinder and squeezed to a pill for determination of energy content in a bomb calorimeter (IKA Calorimeter C7000). Energy uptake is determined as the product of food consumed and the caloric value of the food. To obtain metabolizable energy (E_met_) the energy loss via feces and urine (2% of E_up_; ) were calculated and subtracted from energy uptake. For statistical analysis, all values are presented as means ± SD. Two-way-ANOVA (SigmaStat, Jandel Scientific) was used to test for effects of the factors strain and sex. A linear model with body mass as covariate was applied for the comparison of genotype effects on food intake and metabolisable energy intake. Because mass loss during food deprivation strongly depended on initial body mass this variable was included as covariate in a linear model to analyse compensatory mass reduction in response to the challenge.

**4. RNA isolation and transcriptome analysis of the whole hippocampus**

Male wild type (n=10) and UBB^+1^ tg (n=13) mice of 9 months of age were decapitated after which brains were rapidly macroscopically dissected. The obtained hippocampi of both hemispheres were subsequently frozen separately in liquid nitrogen and stored at -80^o^C until further processing. One hippocampus was used for transcriptome analysis while the contralateral hippocampus was used for proteome analysis. RNA was extracted from a single hippocampus with a standard TRIZOL® (Invitrogen) and chloroform RNA extraction protocol. The integrity of the extracted RNA was assessed with a Bioanalyzer (Agilent, Santa Clara, CA) using the Agilent RNA 6000 Nano Kit. The RNA integrity number had to be at least 7.0 for the RNA sample to be used for cDNA synthesis. The cDNA synthesis was done by using Invitrogen SuperScript II Reverse Transcriptase kit (Invitrogen, Carlsbad, CA). We selected 33 genes based on their role in Alzheimer’s disease, UBB^+1^ processing, the UPS, autophagy and pre- and post-synaptic functioning (Table S4). We also analyzed a miscellaneous group of genes of interest which may play a role in other neurodegenerative diseases or are regulated by UBB^+1^. Primers for all 33 genes of interest and 3 housekeeping genes (Table S5) were designed with Primer3 Plus . All primers are intron spanning and produce a PCR-product with a size of 70 to 120 base pairs with specificity determined by BLAST analysis. The experiments were performed with a LightCycler® 480 and the LightCycler® 480 SYBR Green I Master kit (Roche). A validated assumption-free analysis of qPCR data based on the actual PCR efficiencies was used to calculate mRNA expression levels of all genes . A selection of five candidate housekeeping genes was made after which we applied the GeNorm program for calculating the most reliable housekeeping genes within this selection. The analysis revealed that 1) **hypoxanthine-guanine phosphoribosyl-transferase** (HPRT1), 2) 40S ribosomal protein S27a (RS27α) and 3) glyceraldehyde-3-phosphate dehydrogenase (GAPDH) were the most stable housekeeping genes. The geometric mean of the expression levels of HPRT1, RS27α and GAPDH for each sample was calculated to serve as a normalization factor. Statistical analyses were performed using GraphPad Prism (GraphPad, La Jolla, CA). Differences between strains were evaluated by the Mann-Whitney test. Statistical significance was reached at p<0.05. Data are presented as average values ± standard error of the mean (SEM).

**5. Immunoblot analysis of synaptic markers from hippocampus tissue**

The contralateral hippocampi (see section 4) were homogenized in lysis buffer (0.1% SDS, 0.1% Triton-X100, 1% glycerol, 1 mM EDTA, 1 mM EGTA, 1 mM Na_3_VO_4_, 30 mM NaF and 1x Complete protease inhibitor cocktail (Roche)) after which total protein was assessed with the Bradford protein assay (Bio-Rad). The concentration of total protein was equalized for all samples after which equal volumes of samples were loaded on an acrylamide gel for SDS-PAGE. Subsequently, the proteins were transferred to a nitrocellulose membrane (Bio-Rad) and immunoblotted for the following synaptic markers: glutamate [NMDA] receptor subunit epsilon-1 (GRIN2A/NR2A, Millipore, Billerica MA), **synaptosomal-associated protein 25 (SNAP25, Millipore**, Billerica MA**),** ionotropic glutamate receptor 2/3 (GLUR2, Millipore, Billerica MA), gamma-aminobutyric acid type B receptor subunit 2/3 (GABRB2, clone BD-17, Millipore, Billerica MA) and calcium/calmodulin-dependent protein kinase type II subunit alpha (CaMKIIa, Millipore, Billerica MA). Membranes were also immunoblotted for glyceraldehyde-3-phosphate dehydrogenase (GAPDH, clone 6C5, Fitzgerald, Acton, MA) as a loading control. Antibodies recognizing the synaptic markers were diluted 1:1,000 whereas the antibodies recognizing GAPDH were diluted 1:500,000. Primary antibodies were recognized by IRDye labeled secondary antibodies (Rockland, Gilbertsville, PA) which were diluted 1:10,000 and detected by using multi-fluorescence scanning with the Odyssey® system (LI-COR, Lincoln, NE). Statistical analyses were performed using GraphPad Prism (GraphPad, La Jolla, CA). Protein differences were evaluated by Mann-Whitney test. Statistical significance was set at p<0.05. Data are presented as average values ± standard error of the mean (SEM).

**6. Microarray analysis of the CA3 region**

Animals were sacrificed following a carbon dioxide asphyxiation protocol and the hippocampus was macroscopically dissected from UBB^+1^ tg and control mice at the age of 18 months (5-6 replicates). Whole brains were isolated and immediately frozen in liquid nitrogen cooled isopentane. Brain cryosections (35 µm) were fixed and stained according to a standard protocol provided by Zeiss (Germany). Briefly, sections were incubated in 75% ethanol (2 min, -20°C), stained with cresyl violet (Sigma), and destained in 100% ethanol. A PALM laser-microdissection unit was used to cut out the CA3 region of the hippocampus. Total RNA was isolated with the RNeasy Micro kit (Qiagen) including DNase treatment and only high quality RNA (RIN>7 on an Agilent Bioanalyzer) was used further. Total RNA (5 ng) was amplified using the MessageAmp II-biotin kit (first round) and the MessageAmp II-biotin Enhanced kit (second round). Amplified aRNA (10 µg) was hybridized on Affymetrix GeneChip® MOE 430 A/B arrays containing about 45 k probe sets. Staining and scanning was done according to the Affymetrix expression protocol. Data was analyzed with ChipInspector (Genomatix, ) using standard settings. Results were confirmed by Bioconductor tools implemented in CARMAweb (RMA, Limma t-test, FDR<10%; ). The probe set 1427508_at was excluded from the analysis, since it detected a transcribed part (but not protein coding) of the CamKIIα promoter used for transgene expression. Statistically significantly regulated genes were further analyzed using the Ingenuity Pathway software and heat maps were generated with the tools provided by CARMAweb. Array data has been submitted to the Gene Expression Omnibus database (GSE13691).

**Supplementary results**

**1. Behavioral changes**

Behavioral analysis of spontaneous activity in a novel environment was measured by the modified Hole Board test . In mice at the age of 3 months, the path shape analysis revealed that UBB^+1^ tg mice of both sexes travelled with fewer changes in direction of movement, as measured by lower turn angles and less meandering (Fig. S8A) compared to the wild type control group. However, no differences were found in transgenic mice with regard to the remaining parameters of forward locomotion such as line crossings, total distance travelled and velocities (data not shown). With respect to the exploration of the familiar and the unfamiliar object present in the arena during the test (for details see supplementary methods), UBB^+1^ tg mice explored the familiar object less than wild type animals, which was significantly more pronounced in female mutants (Fig. S8A; genotype effect: F _(1, 56)_ = 5.675, p < 0.05; genotype x sex interaction: F _(1, 56)_ = 4.277, p < 0.05). Independent of the sex, UBB^+1^ tg mice showed a trend to explore the unfamiliar object more intensively than control animals (Fig. S8A; genotype effect: F _(1, 56)_ = 3.203, p = 0.08). This pattern produced a positive object recognition index in mutants, in contrast to controls (0.07 ± 0.06 vs. -0.19 ± 0.06; p < 0.01).

The analysis of mice at the age of 12 months (Fig. S8B) revealed no genotype effect on locomotion-related parameters, but UBB^+1^ tg mice travelled a greater distance to the board, which represents an exposed area in the middle of the test apparatus, possibly indicating increased anxiety. Regarding vertical exploration, UBB^+1^ tg mice of both sexes exhibited rearings to the board later than controls, but the total number of rearings was the same (Fig. S8B and data not shown). We also observed changes in object exploration with UBB^+1^ tg mice of both sexes spending less time with the familiar object than controls (Fig. S8B; genotype effect: F_(1,56)_ = 4.783, p < 0.05). There were no genotype effects on any other measured/studied parameter.

The changes in path shape which were observed in UBB^+1^ tg mice at the age of 3 months were not present in older animals. Concerning object exploration, like the younger mutants the older ones also explored the familiar object less than controls. However, object memory, indicated by a positive object recognition index, did not differ between UBB^+1^ tg mice and controls at 12 months.

In addition to the modified Hole Board Test, we studied a hippocampus-dependent learning paradigm, i.e., the spontaneous alternation in the Y-maze that represents a measure of spatial working memory (, Fig. S8C/D). UBB^+1^ tg mice at the age of 6 months revealed significantly reduced levels of spontaneous alternation compared with wild-type controls (60% versus 71%; p<0.05). The total number of arm entries during Y-maze testing was not significantly different between the groups. Therefore, in this test the levels of exploratory activities were not affected.

**2. Metabolic changes**

The monitoring of body mass, food intake and body temperature during *ad libitum* conditions and in response to a food deprivation challenge revealed specific genotype related differences in UBB^+1^ tg mice compared to control mice at three and twelve months of age. During *ad libitum* feeding body mass and body temperature did not differ between control and tg mice at three and twelve months of age whereas food intake and metabolisable energy adjusted to body mass trended to be decreased (3 months old UBB^+1^ tg mice, food intake: genotype p = 0.0533, metabolizable energy: genotype p = 0.0395; 12 months old UBB^+1^ tg mice, food intake: genotype p = 0.0536, metabolisable energy: genotype p = 0.0609, Fig. S9/10). At twelve months of age but not in younger mice the assimilation efficiency calculated as the ratio of energy extracted from food was significantly reduced in UBB^+1^ tg mice (genotype p = 0.0319, data not shown). Mice at this age were also exposed to a 2 days food deprivation challenge, where especially male UBB^+1^ tg mice showed a weaker body mass loss in response to the fasting challenge (7-15% lower, Fig. S11). Differences in the compensatory reduction of body temperature during the fasting challenge could not be detected between wild type and tg mice (data not shown).

**3. Gene expression changes in the hippocampal formation**

Using assumption free quantitative qPCR, we studied the expression in the hippocampal formation of a panel of genes mainly defined by their known role in AD, UPS and pre- or postsynaptic functioning. Of the 35 genes tested, 7 were significantly upregulated in the UBB^+1^ tg mice versus controls. They comprise five proteins involved in synaptic functioning: GRIN2A (151%), CAMK2A (151%), SNAP25 (143%), GABRB2 (134%) and GLUR2 (129%) as shown in Fig. S12A, as well as 14‑3‑3 *zeta (ζ)* 260%) and APP (158%; Fig. S12D). 27 other genes involved in synaptic functioning, AD, UPS. Autophagy, or other functions were not statistical significantly regulated (Supplementary Fig. S13).

We next analyzed the protein levels of those genes, which were regulated on mRNA level, by Western blotting or immunohistochemistry. Contrary to the transcript data most of the synaptic proteins did not show a significant difference at the protein level or were even regulated in the opposite direction in case of GABRB2 (Fig. S12B/C). 14-3-3 zeta (ζ) was the only gene with consistent regulation on transcript and protein levels. Immunohistochemical staining clearly showed increased expression of 14‑3‑3 zeta protein in the CA1 region of the hippocampus (Fig. S12E) and it was significantly upregulated in 22 months old UBB^+1^ tg mice to 186% of normal levels (personal communication Prof. Dr. K. Marcus and Dr. T. Schulenborg, Bochum, data not shown). 14‑3‑3 zeta functions as a heat shock protein and is known to be involved in many processes such as the solubilization of aggregated proteins.

**4. Transcriptomic changes in the hippocampal CA3 region**

In our expression analysis by qPCR of synaptic markers in the whole hippocampus most of the observed changes were below 1.5-fold. This might in part be caused by the cellular heterogeneity of the hippocampal formation where genes might only be regulated in specific subregions making it difficult to detect expression differences. We therefore studied gene regulation in the hippocampal CA3 subregion, which is known to be relevant for memory functions . The CA3 region was isolated by laser-microdissection from histological cryo-sections of the hippocampus and used for transcriptome profiling on Affymetrix MOE 430 A/B arrays. We analyzed RNA derived from mice at the age of 18 months (n=6 for UBB^+1^ tg and n=5 for control mice). The statistical analysis of the array data with the ChipInspector software (Genomatix) identified 274 significantly regulated genes (FDR<10%) in UBB^+1^ tg mice in comparison to controls. Most of the transcripts were upregulated (218) with fold-changes up to about 2-fold (Table S1). To assess the predicted functions of regulated genes, we searched for significantly enriched pathways and functional annotations associated with these genes employing the Ingenuity Pathway Analysis software. The most significantly enriched terms (p<0.05) were related to neuron-specific functions (synaptic transmission, long term depression), UPS, locomotion and behavior of mice, calcium-related processes, exocytosis, and neurological diseases (Table S2). In our microarray analysis we did not observe an increased UBB expression in tissue from UBB^+1^ transgenic mice, most likely because the human transgene was not detected on the mouse arrays. However, we were able to confirm transgene expression by qPCR on whole hippocampus tissue as previously published (data not shown).

**Supplementary discussion**

**1. Behavioral changes and hippocampal gene expression**

The modified Hole Board Test allows the analysis of a range of behavioral parameters such as locomotor activity, exploratory behavior, arousal, emotionality, memory and social affinity in a single short test . This broad approach seemed to be suitable for UBB^+1^ tg mice, where the transgene is expressed in several brain regions (cortex, CA1-3, dentate gyrus, amygdala) known to be involved in these functions. Concerning the memory task within the modified Hole Board Test, mutant mice explored a familiar object less and explored an unfamiliar one more. Thus, young transgenic mice performed better in distinguishing between familiar and unfamiliar objects than control animals as also indicated by the positive object index, suggesting better object recognition memory. However, object memory was not improved in old mutants, but a decreased exploration of the familiar object was the most consistently altered parameter in UBB^+1^ tg mice of both ages. These results indicate that UBB^+1^ tg mice do not suffer from any deficit in object recognition memory. This finding does not necessarily contradict the published cognitive deficits of this mouse line, since those were related to spatial and associative learning, but not to object recognition. The subtle changes in path shape, rearing latency and distance to the board are of unclear relevance as they were not observed in tg mice of both ages. Other parameters reflecting locomotion (line crossings, total distance travelled, velocities, vertical exploration in the box) remained unchanged, indicating that there were no general alterations in locomotor or exploratory activity in the mutants. Also, anxiety levels as measured by time spent on the board were not altered. Taken together, in our behavioral analysis of UBB^+1^ tg mice we observed “subtle” but significant and reproducible changes regarding object exploration. In addition, we also identified an impairment of the hippocampus-dependent spontaneous alternation in the Y-maze test. In this regard it was interesting to see that our transcriptome analysis of the hippocampal CA3 region suggests changes in in regulation of genes, which could be directly linked to the observed phenotypic data (Table S2). Regulated transcripts are known to be involved in behavior-related functions (memory, locomotion, and conditioning) as well as general brain functions (synaptic transmission, long term depression). Also our gene expression analysis of whole hippocampus tissue (Fig. S12) indicates the significant regulation of genes related to synaptic functioning. Our new data therefore extend the recent findings in UBB^+1^ tg mice and provide potential explanations at a molecular level.

**2. Metabolic changes**

We measured bioenergetic parameters in the context of body weight regulation and energy metabolism during *ad libitum* feeding and under food restricted conditions. We observed hypophagia under *ad libitum* feeding and a weaker response to the fasting challenge in UBB^+1^ mice. The observed effect of UBB^+1^ expression on bioenergetic parameters and gene expression is supported by previously published proteomic studies, where differential expression or modification of proteins related to energy metabolism was observed in the cortex of UBB^+1^ tg mice or in brains of AD patients or mouse models for AD . However, we did not observe any weight loss, which is known to precede diagnosis of AD, PD, and Huntington in humans and which has been published for several mouse models for AD . In contrast, Ubb-deficient mice have metabolic and sleep abnormalities leading to adult-onset obesity . This phenotype is caused by hypothalamic neurodegeneration and is reminiscent of an altered circadian rhythmicity, which is known to be tightly linked to metabolism . In our mouse model, UBB^+1^ protein was not detected in the hypothalamus and we did not observe any neurodegeneration in the brain. Given the importance of the UPS for the regulation of clock proteins , we cannot rule out the possibility that the partial proteasome inhibition by UBB^+1^ affects the circadian clock in distinct parts of the brain and thereby causes the observed metabolic changes.

**3. Transcriptomic and proteomic changes in the hippocampus**

Although the primary consequences of UPS inhibition take place on the protein level, these changes might also affect the transcript level. In line with this, we were able to identify regulated genes and biological functions in our analyses of whole hippocampus and the hippocampal CA3 region. In the whole hippocampus we observed the upregulation of 14‑3‑3 ζ in UBB^+1^ tg mice, both at transcript and protein levels. 14-3-3 ζ is mainly present in the CA1 region of the hippocampus and was not found to be differentially expressed in our analysis of the CA3 region. The 14-3-3 ζ protein is known to be a heat-inducible molecular chaperone which is able to refold proteins after heat shock induction . The molecular mechanism as to how UBB^+1^ which does not aggregate itself induces a heat shock protein remains to be determined but it is known that UBB^+1^ is able to induce the expression of heat shock proteins . Other upregulated transcripts were those for APP and genes involved in synaptic functioning. Most of these changes were below 1.5-fold, which might have hindered their detection at the protein level by western blotting. Another possible explanation for the poor correlation between our mRNA and protein levels might be post-translational modifications, which have been described previously for many proteins .

The UBB^+1^ protein is (poly)ubiquitinated by E2-25K/Ube2k and the E3 ligase TRIP12 . In addition, the C-terminus of UBB^+1^ is hydrolyzed by ubiquitin C-terminal hydrolase UCH-L3 . Although clearly involved in UBB^+1^ processing, none of these proteins was differentially expressed in hippocampal tissue of UBB^+1^ tg animals. However, we observed a significant enrichment of UPS-associated genes in our transcriptome analysis of the CA3 region (Table S3). The 16 UPS-related genes (Psmc6, Psmc5, Xiap, Usp45, Ube3a, Usp10, Usp14, Brwd1, Mocs2, Uba3, Ddi2, March7, Pcmtd2, Rspry1, Tbcel, and Trim2) could be grouped according to their function in ligases (8 genes), proteases (3), proteasomal subunits (2) and ubiquitin-like proteins (3). Although the precise functions and molecular targets of most genes are unknown, twelve of the genes have been previously discussed in the context of neurological diseases (Table S3). For example, loss of Ube3a causes Angelmann syndrome, which is associated with learning deficits and impaired long-term potentiation and mice deficient in the ubiquitin ligase Trim2 or the protease Usp14 develop ataxia. Usp10 has been associated with an increased risk for glioblastoma and was recently shown to directly mediate the stability of p53. The fact that most of the UPS genes were upregulated is suggestive of a compensatory effect. *The* short-term inhibition of the proteasome has been reported to result in a strong induction of proteasomal subunits, which we did not observe. In fact, the only downregulated UPS gene in our dataset was the proteasomal ATPase Psmc5. It degrades ataxin-3 in Machado-Joseph disease and is known to be down regulated by long-term depression in humans .

**Supplementary figures**

Fig. S1. UBB^+1^ affects spontaneous breathing patterns measured by whole body plethysmography.

Female UBB^+1^ tg (black bars) and control mice (grey bars) were analyzed at 12 months of age (n=6 for each group). Shown are the mean expiratory flow rate (MEF), expiratory time (Te), relative duration of inspiration (Ti/TT), and the mean inspiratory and expiratory flow rate (MIF, MEF) under rest and activity, respectively. The mean values for the indicated parameters of the control group were set to 100% and significance by Student´s t-test is indicated by * p<0.05; error bars represent SEM.


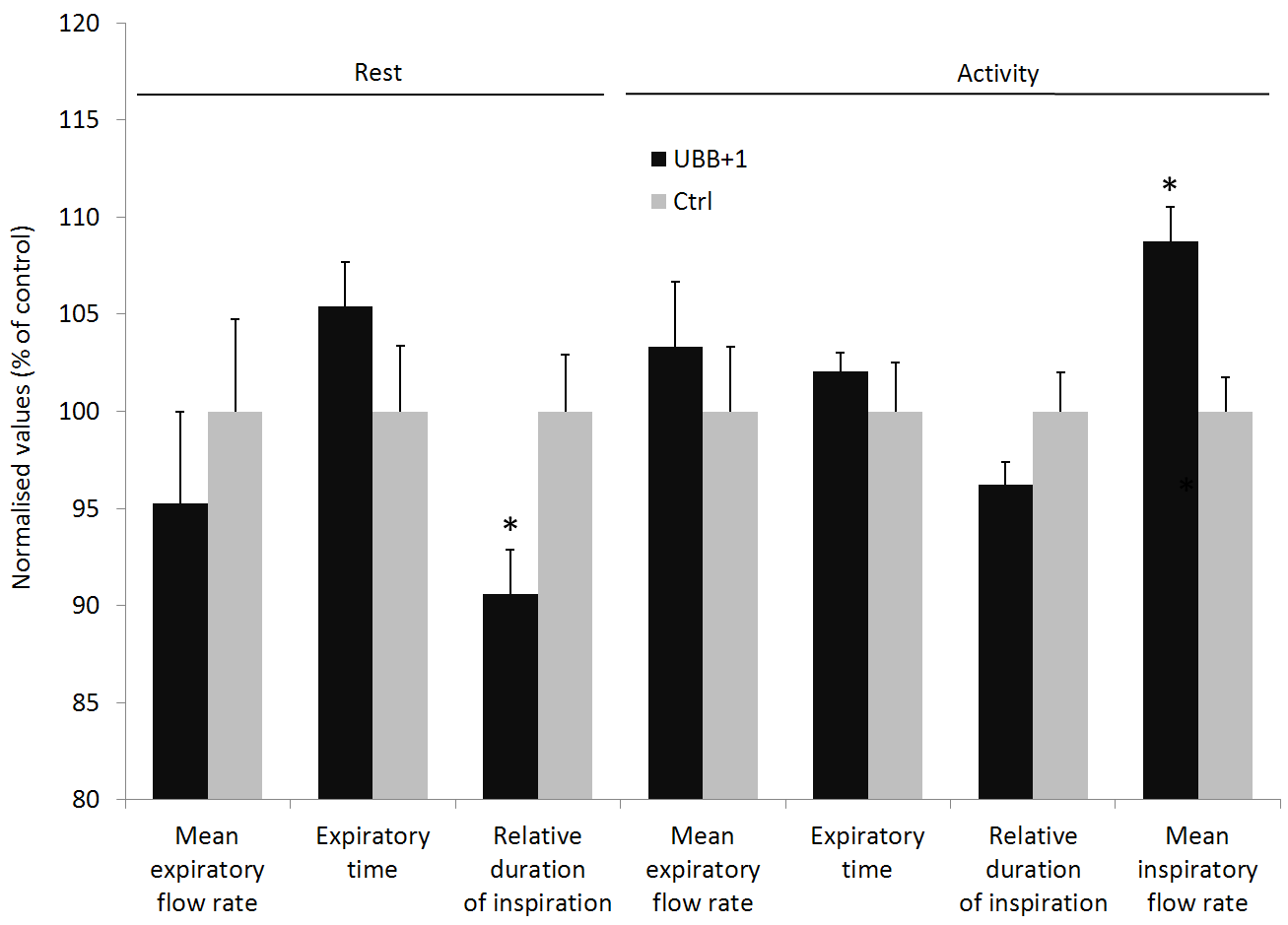


Fig. S2. UBB^+1^ affects spontaneous breathing patterns measured by whole body plethysmography.

Male UBB^+1^ tg (black bars) and control mice (grey bars) were analyzed at 18 months of age (n=8 for each group). Shown are the minute ventilation (MV), specific ventilation (sMV), peak expiratory flow rate (PEF), and the mean expiratory flow rate (MEF) under rest and activity, respectively. The mean values for the indicated parameters of the control group were set to 100% and significance by Student´s t-test is indicated by * p<0.05; error bars represent SEM


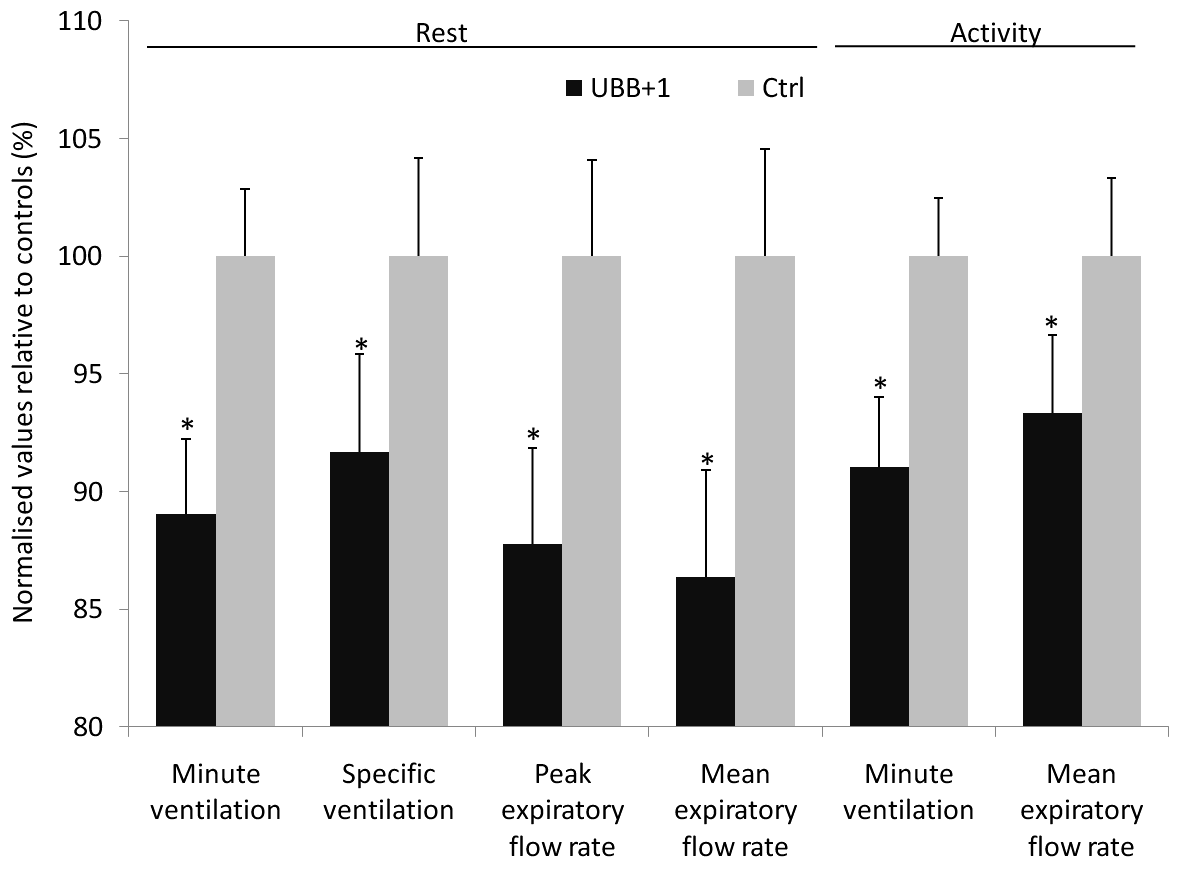


Fig. S3. Lung function of male UBB^+1^ tg mice at the age of 18 months is not altered. To ensure that differences in breathing pattern are not caused by alterations of the lung a comprehensive lung function test was carried out (n=8 for each group). In comparison to controls none of the measured parameters was significantly affected in UBB^+1^ mice. This indicates that differences in lung size (TLC) or its subdivisions (IRC, FRC, ERV, VD) cannot account for the observed differences in breathing pattern. Similarly, measures of the mechanical properties including the compliance of the respiratory system and the lung under static (C, C_L_) as well under dynamic conditions (C_dyn_, C_dynL_) and the resistance of the respiratory system cannot provide a reasonable explanation. Also the gas exchanging capacity of UBB^+1^ lungs was not affected by the mutation as shown by comparable values for the slope of the alveolar plateau of the helium expirogram (S_He_) - a measure of the quality of intrapulmonary gas transport and mixing - and for the diffusing capacity of carbon monoxide (D_CO_, D_CO_/TLC) - a measure of the extent of alveolar capillary gas transfer into the pulmonary circulation.


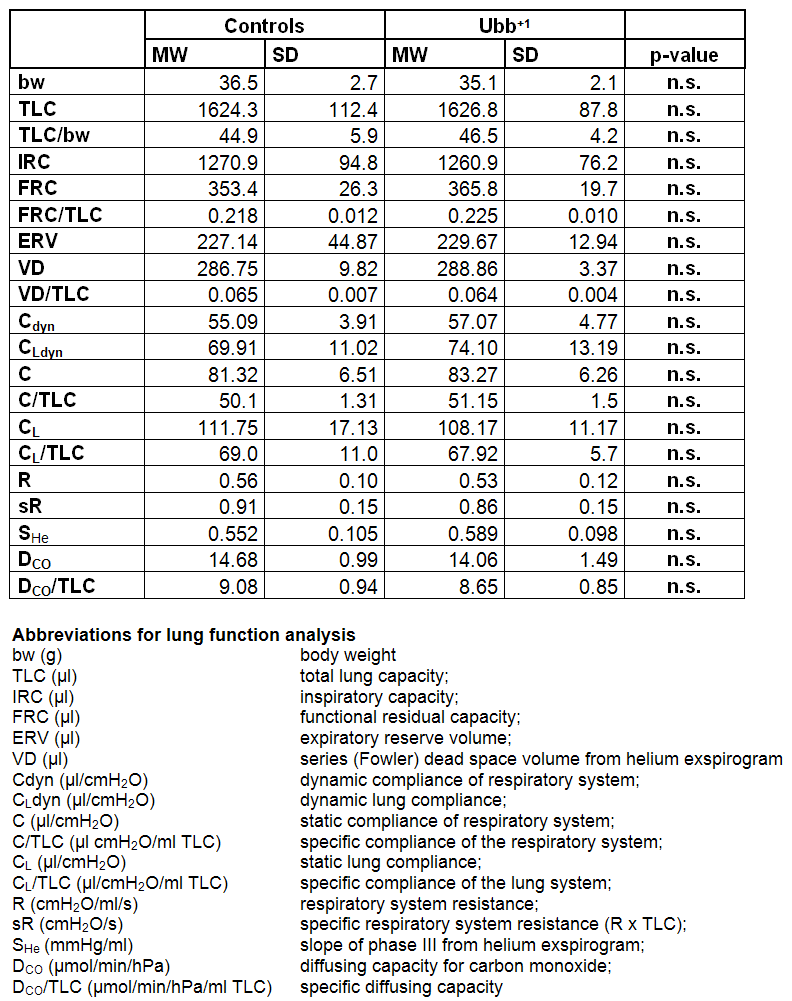


Fig. S4. Data for spontaneous breathing pattern of UBB^+1^ tg mice at the age of 3 months during rest and activity. n.s., not significant; abbreviations are provided in the supplementary methods

**
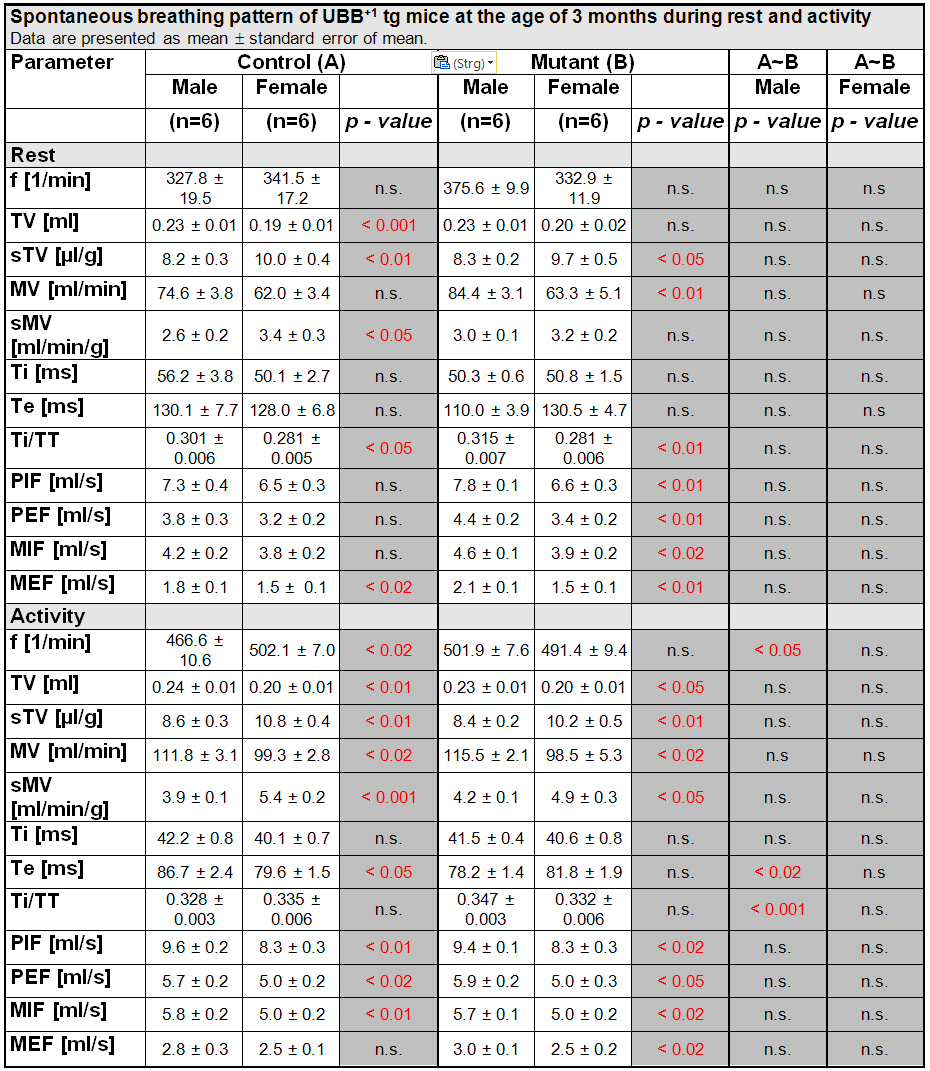
**

Fig. S5. Spontaneous breathing pattern of UBB^+1^ tg mice at the age of 12 months during rest and activity. n.s., not significant; abbreviations are provided in the supplementary methods

**
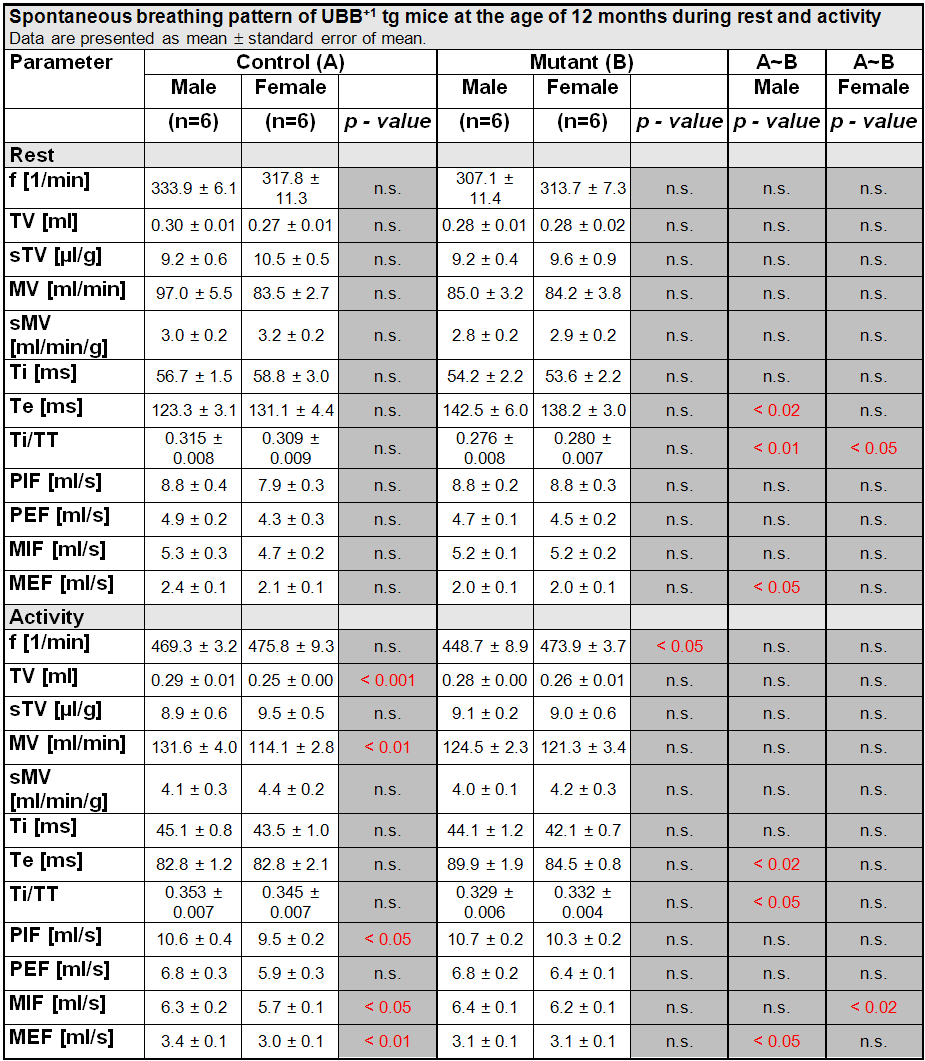
**

Fig. S6. Spontaneous breathing pattern of UBB^+1^ tg mice at the age of 18 months during rest and activity. n.s., not significant; abbreviations are provided in the supplementary methods


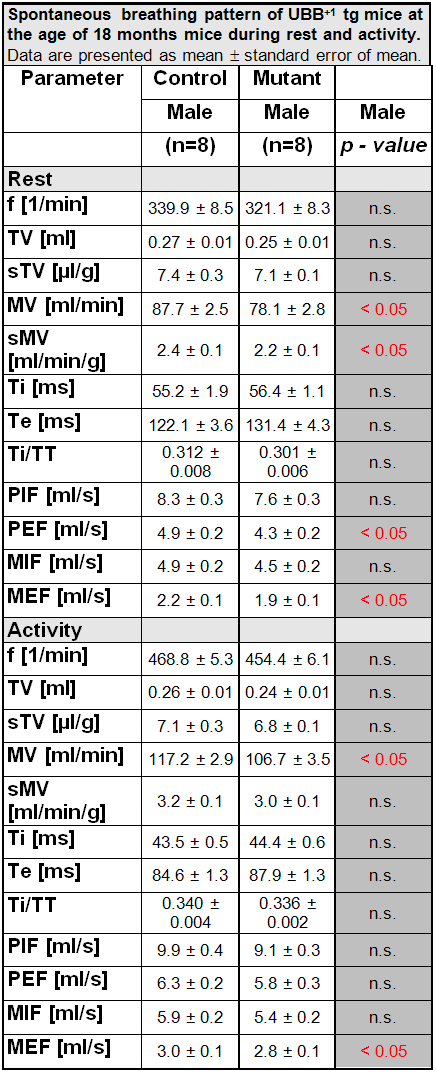


Fig. S7. Ventilatory response to hypercapnic conditions is not affected by UBB^+1^ expression. Male mice at the age of 18 months (n=8 for each group) were exposed to 3/5/8% CO_2_ (hypercapnia) for 7 minutes each followed by a recovery period of 15 minutes. (a) Shows the respiratory rates (f), (b) shows the tidal volume (TV), (c) shows the minute ventilation (MV), and (d) shows the relative duration of inspiration time (Ti/TT). Values were calculated relative to baseline levels (set to 100%).


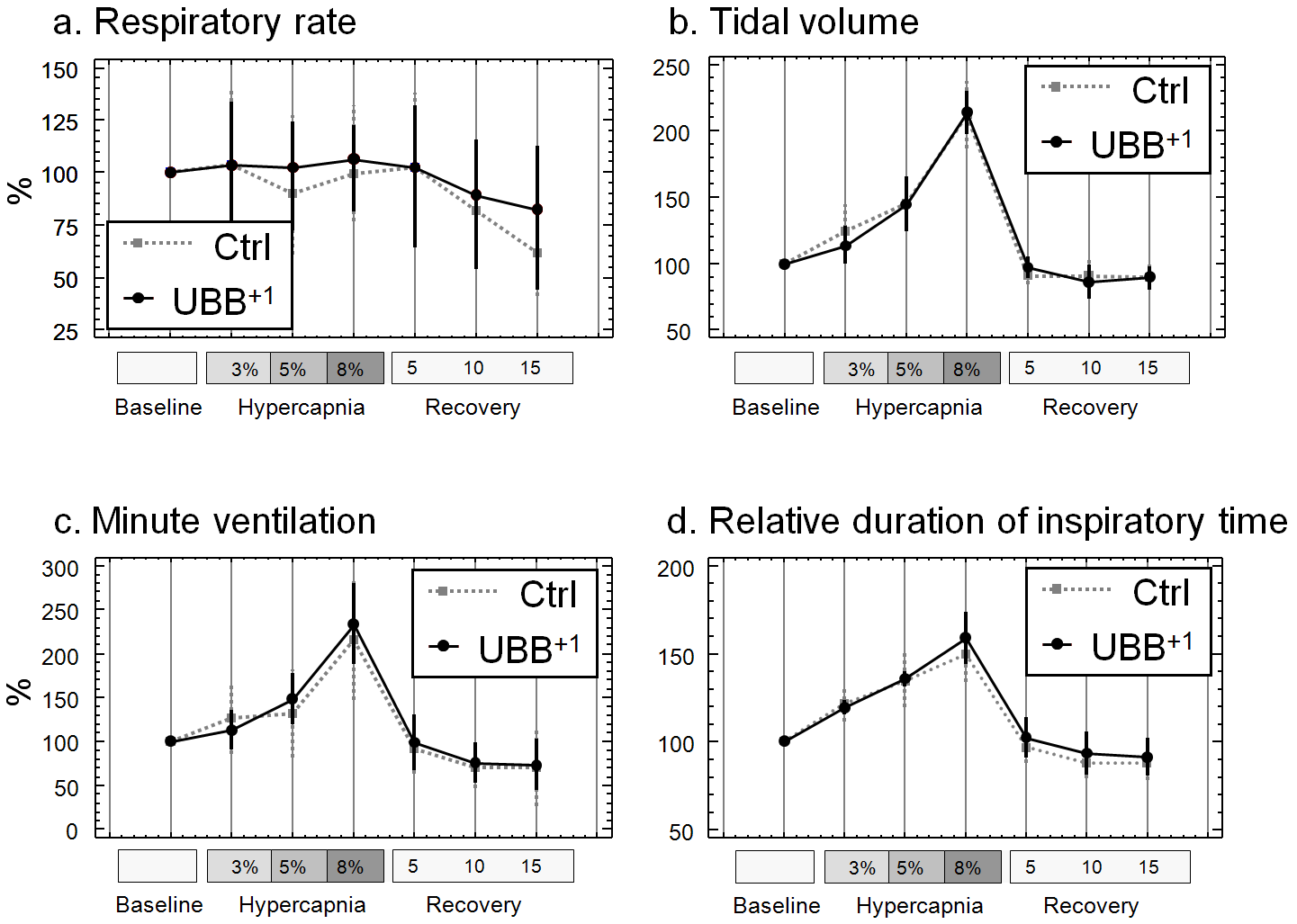


Fig. S8. UBB^+1^ tg mice behave differently than wt mice. Modified Hole Board analysis revealed significant differences between UBB^+1^ transgenic animals and controls at the age of (a) 3 months and (b) 12 months. Spatial working memory of mice at 6 months of age was tested using the spontaneous alternation Y-maze task (c/d). Spatial working memory, as assessed by the spontaneous alternation performance is significantly impaired in UBB^+1^ mice compared to wild-type controls (c). Total number of arm entries reflecting exploratory activity of mice in the Y-maze does not show a significant difference (d). Student´s t-test,

* p<0.05, ** p<0.01; error bars represent SEM.


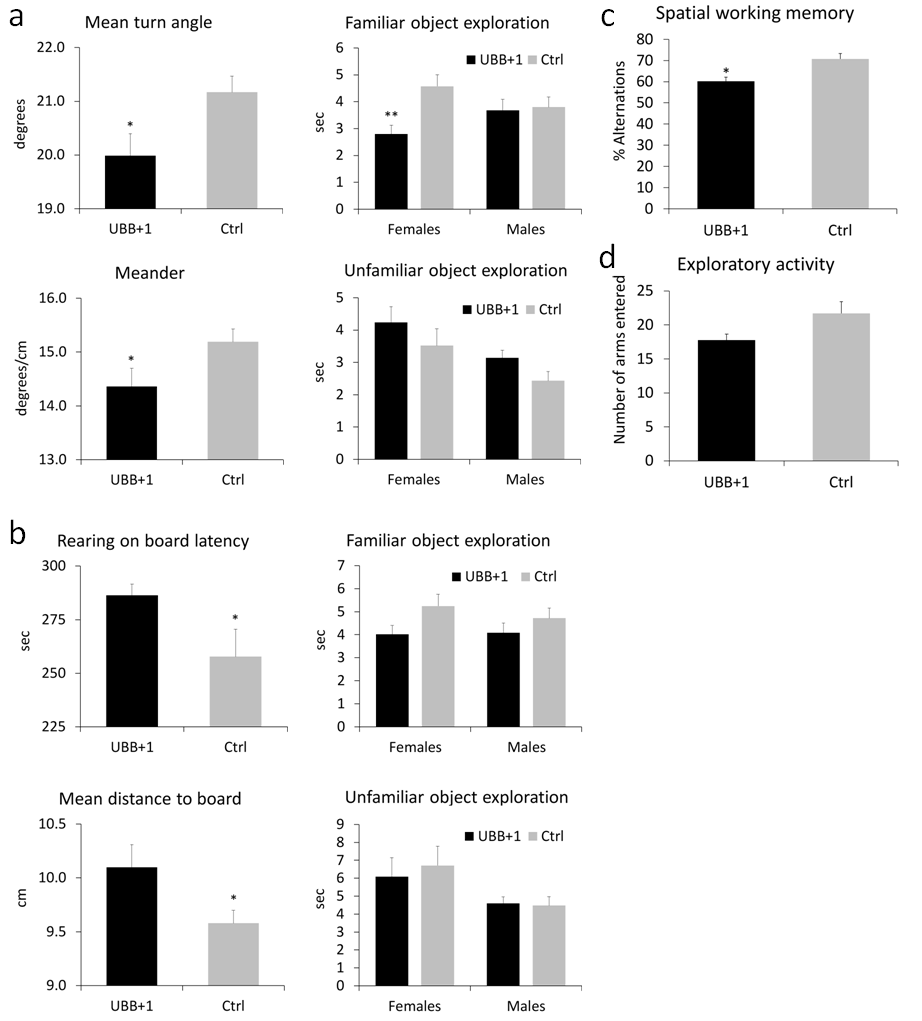


Fig. S9. UBB^+1^ tg mice at three months of age trended to show a significantly lower food intake than control animals (genotype p = 0.0533, linear regression model with body mass as covariate). The effect missed significance when sexes were tested separately. A similar trend was found in UBB^+1^ tg mice in males at the age of twelve months (genotype p = 0.0536).


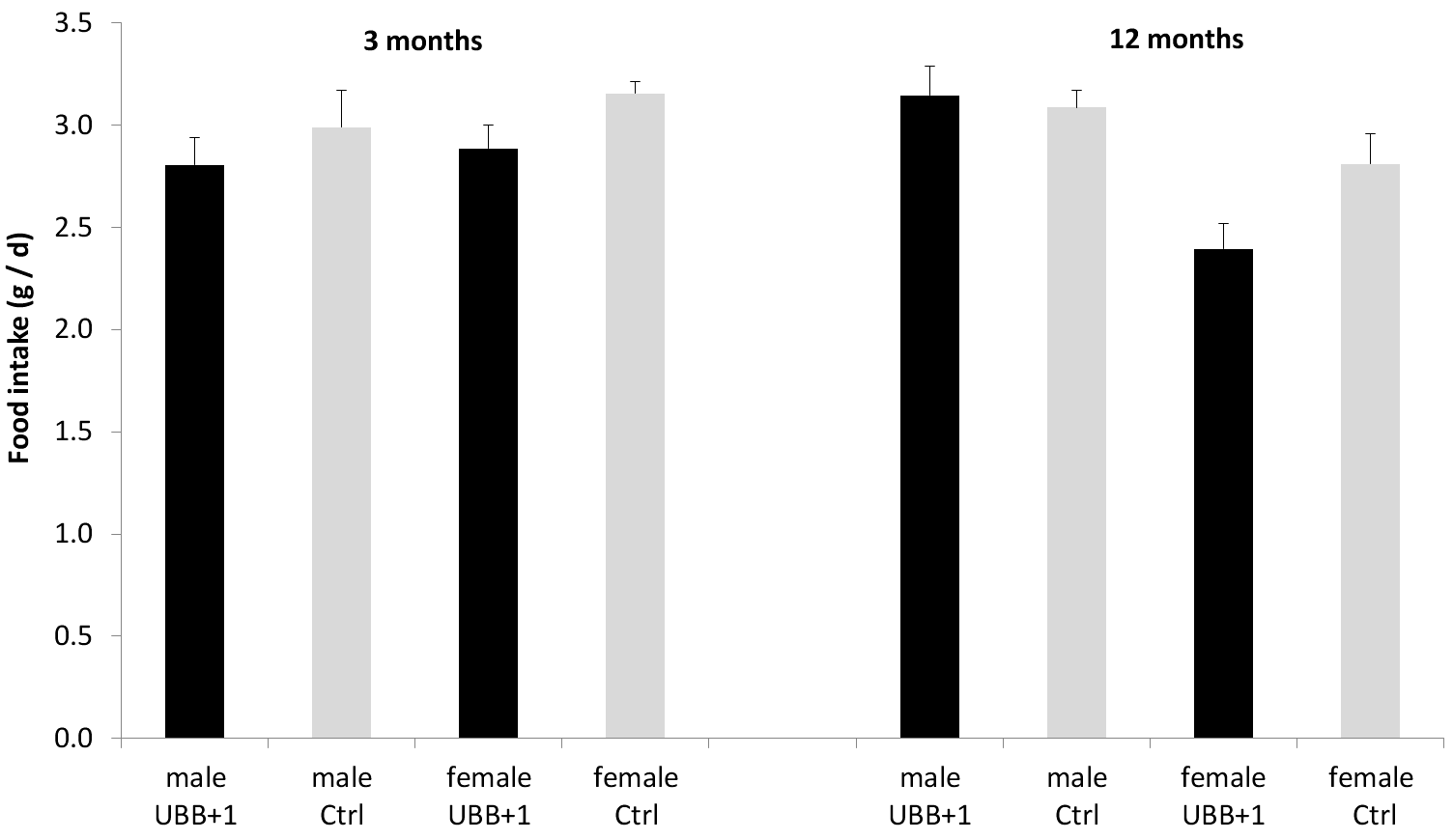


Fig. S10. UBB^+1^ tg mice at three months of age trended to show a significantly lower amount of metabolisable energy than control animals (genotype p = 0.0395, linear regression model with body mass as covariate). The effect missed significance when sexes were tested separately. A similar trend was found in UBB^+1^ tg mice at the age of twelve months (genotype p = 0.0609).


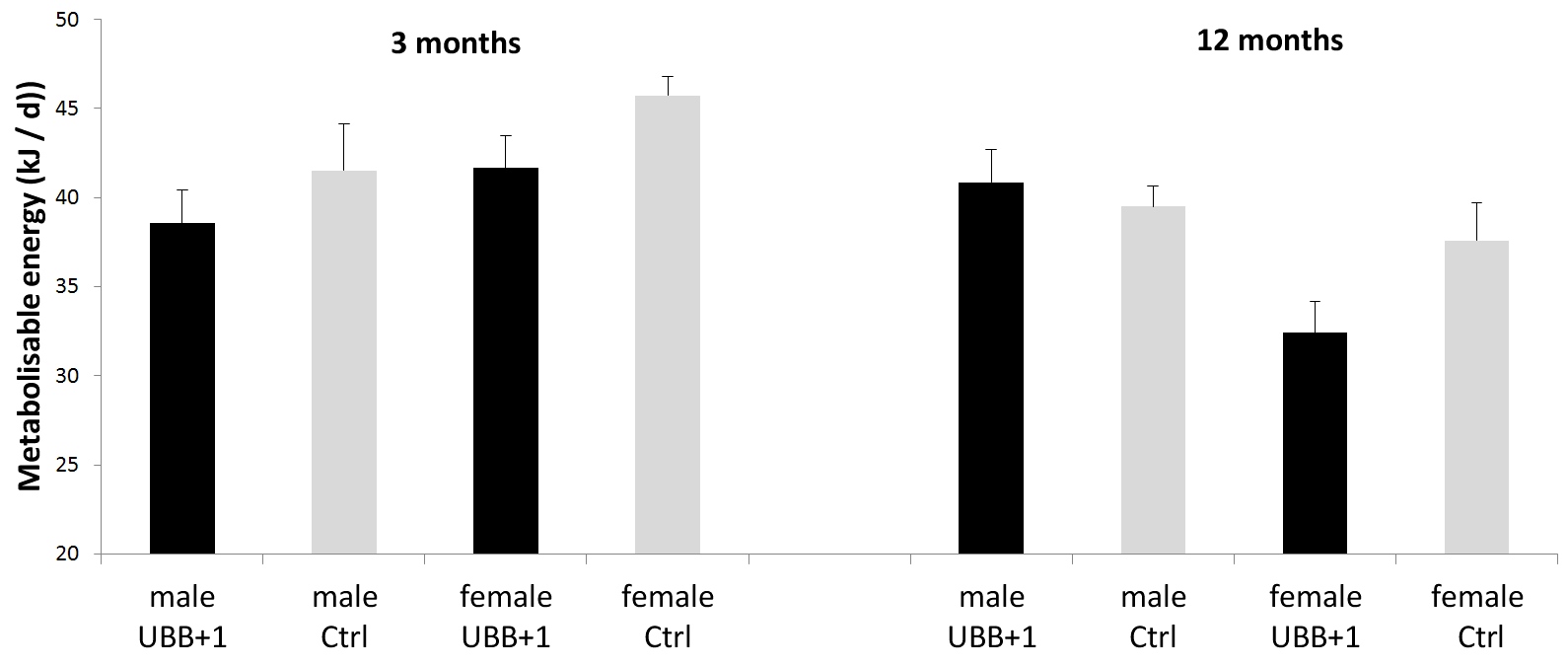


**Fig. S11. Diminished weight loss in UBB^+1^ tg mice upon a food deprivation challenge.** A food deprivation challenge for two days resulted in a diminished weight loss in 12 months old male UBB^+1^ tg mice compared to control animals (p<0.05). Female tg and control mice behaved similarly.


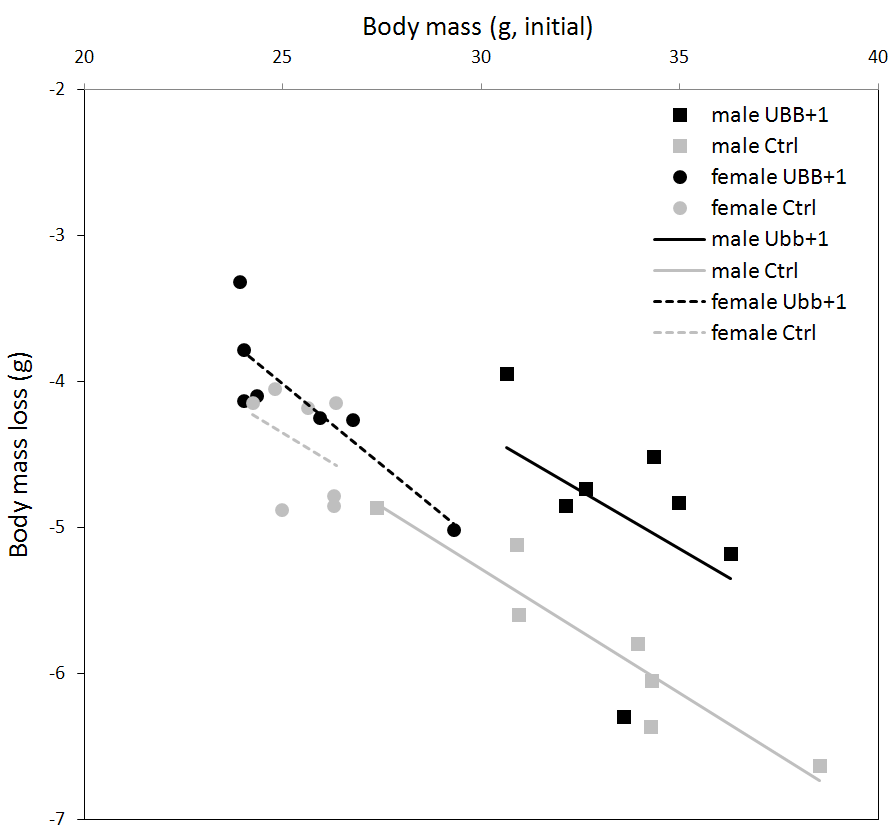


**Fig. S12. Regulation of transcript and protein levels in the hippocampus .** Synaptic marker gene profiles (a/b/c) and 14-3-3 ζ expression (d/e) in tg and control animals at the age of 9 months. Differentially regulated genes in the hippocampus of UBB^+1^ tg animals versus age matched controls at mRNA (a) and protein levels (b/c). Note that an increase in gene expression does not automatically result in an increase in protein expression (e.g. for GABRB2). mRNA profile (d) and protein expression by immunohistochemistry (e) of 14‑3‑3 ζ. Arrowheads indicate the pyramidal cell (e.g., CA1) region. Note the significant increase in the mRNA expression for 14-3-3 ζ (p<0.01). In addition the mRNA of APP was increased significantly as a result of UBB^+1^ overexpression. Error bars represent SEM. *p<0.05, **p<0.01

**
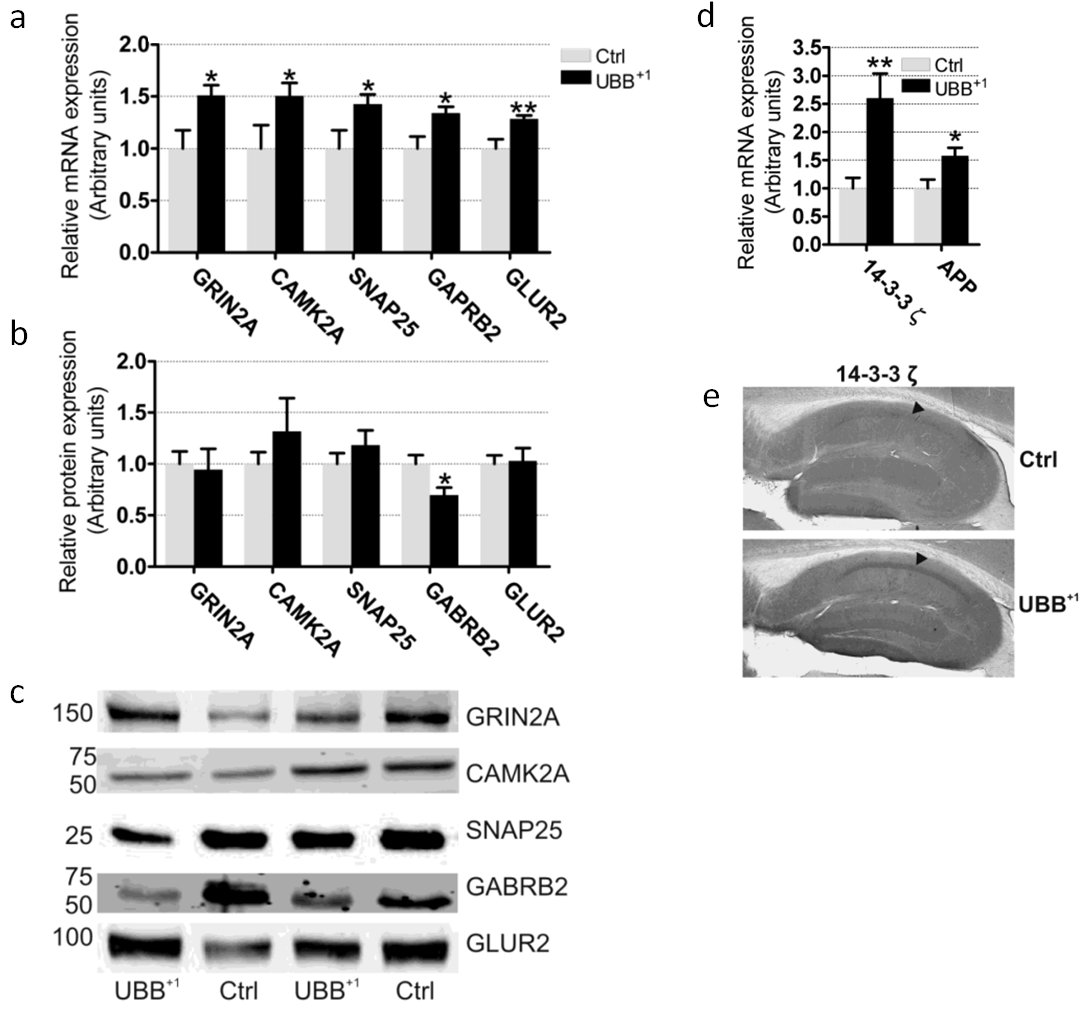
**

**Fig. S13. Genes not significantly regulated in UBB^+1^ tg animals versus controls.** Controls (open boxes), UBB^+1^ tg animals (filled boxes)


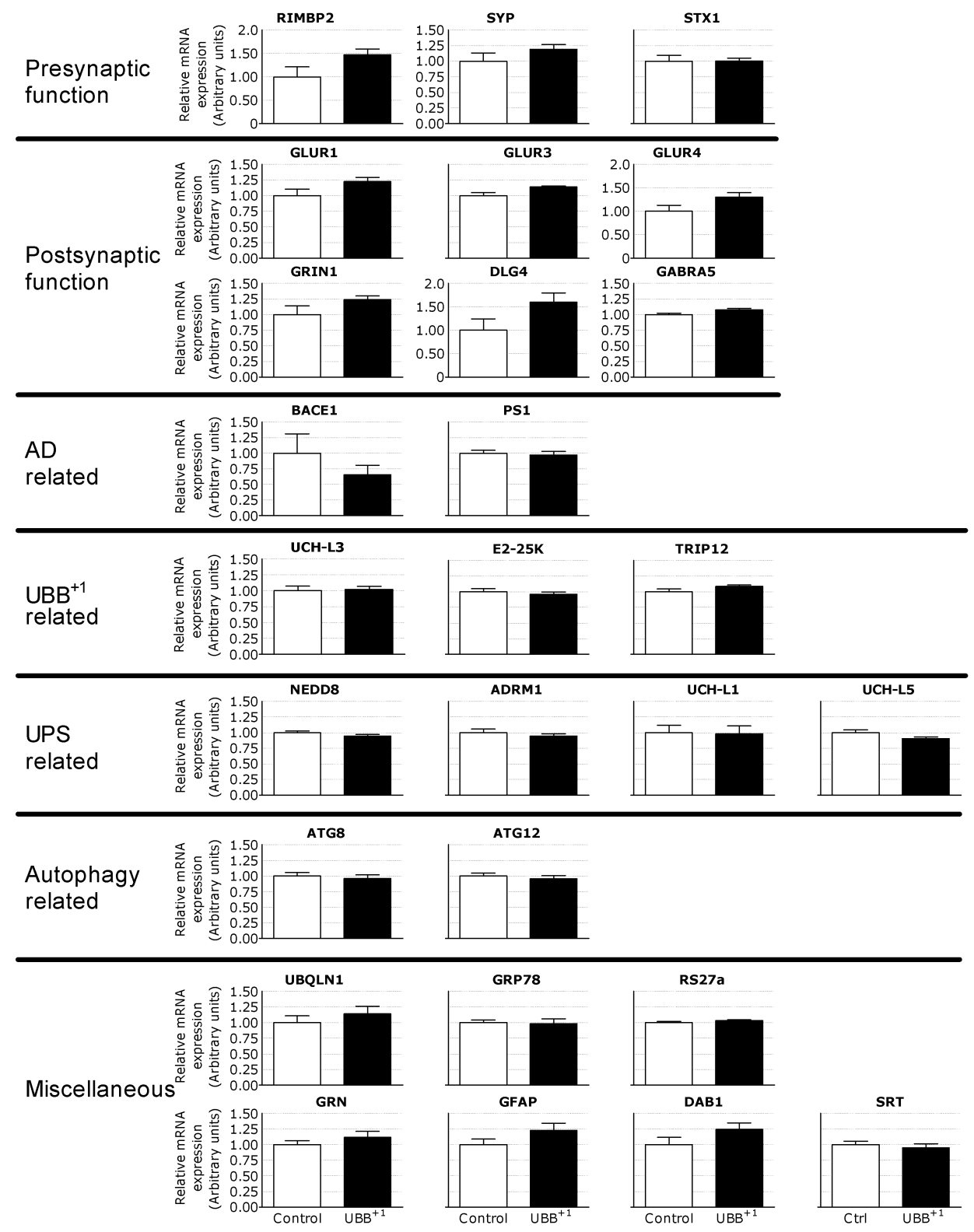


Supplementary tables

**Table S1. 274 genes are significantly (FDR<10%) regulated in the CA3 region of UBB^+1^ tg mice compared to control animals**

| **Gene symbol** | **Fold-change (linear)** | **Entrez ID** | **Accession Number** | **Gene name** |
| --- | --- | --- | --- | --- |
| Slmap | 1.9 | 83997 | AK146685 | sarcolemma associated protein |
| Hbp1 | 1.8 | 73389 | AK136633 | HMG-box transcription factor 1 |
| Zyg11b | 1.8 | 414872 | AK034007 | zyg-11 homolog B (C. elegans) |
| Zfp445 | 1.8 | 235682 | AK036132 | zinc finger protein 445 |
| Ogfrl1 | 1.7 | 70155 | AK034467 | opioid growth factor receptor-like 1 |
| Cntnap2 | 1.7 | 66797 | NM_025771 | contactin associated protein-like 2 |
| Gm5578 | 1.7 | 434077 | XM_485815 | predicted gene 5578 |
| 0610010K06Rik | 1.7 | 71678 | AK050525 | RIKEN cDNA 0610010K06 gene |
| Cadps | 1.7 | 27062 | NM_001042617 | Ca++-dependent secretion activator |
| Rapgef4 | 1.7 | 56508 | ENSMUST00000028525 | Rap guanine nucleotide exchange factor (GEF) 4 |
| Gdap1 | 1.7 | 14545 | NM_010267 | ganglioside-induced differentiation-associated protein 1 |
| Mtpn | 1.7 | 14489 | AK077293 | myotrophin |
| Larp5 | 1.7 | 217980 | AK148903 | La ribonucleoprotein domain family, member 5 |
| Aqp4 | 1.7 | 11829 | AK079614 | aquaporin 4 |
| Cacnb4 | 1.7 | 12298 | ENSMUST00000102761 | calcium channel, voltage-dependent, beta 4 subunit |
| Serinc1 | 1.7 | 56442 | AK003749 | serine incorporator 1 |
| Pbrm1 | 1.7 | 66923 | ENSMUST00000090214 | polybromo 1 |
| Clps | 1.7 | 109791 | NM_025469 | colipase, pancreatic |
| St8sia6 | 1.7 | 241230 | AK156557 | ST8 alpha-N-acetyl-neuraminide alpha-2,8-sialyltransferase 6 |
| Nfib | 1.7 | 18028 | NM_001113210 | nuclear factor I/B |
| Tmeff1 | 1.7 | 230157 | AK079633 | transmembrane protein with EGF-like and two follistatin-like domains 1 |
| LOC100045217 | 1.7 | 100045217 | XM_001473880 | similar to Transmembrane protein with EGF-like and two follistatin-like domains 1 |
| Wasl | 1.7 | 73178 | AK013566 | Wiskott-Aldrich syndrome-like |
| Ube3a | 1.7 | 22215 | NM_001033962 | ubiquitin protein ligase E3A |
| March7 | 1.7 | 57438 | AK080468 | membrane-associated ring finger (C3HC4) 7 |
| Pfkfb3 | 1.7 | 170768 | ENSMUST00000028114 | 6-phosphofructo-2-kinase/fructose-2,6-biphosphatase 3 |
| Chm | 1.7 | 12662 | ENSMUST00000113388 | choroideremia (Rab escort protein 1) |
| LOC100046205 | 1.7 | 100046205 | XM_001475778 | similar to choroidermia |
| Wdfy1 | 1.7 | 69368 | ENSMUST00000097685 | WD repeat and FYVE domain containing 1 |
| Paics | 1.7 | 67054 | AK075949 |  |
| 6330406I15Rik | 1.7 | 70717 | NM_027519 | RIKEN cDNA 6330406I15 gene |
| Ahcyl2 | 1.7 | 74340 | NM_021414 | S-adenosylhomocysteine hydrolase-like 2 |
| Fam40b | 1.7 | 320609 | NM_177204 | family with sequence similarity 40, member B |
| Hdgfrp3 | 1.7 | 29877 | AK012267 | hepatoma-derived growth factor, related protein 3 |
| Sstr2 | 1.7 | 20606 | ENSMUST00000106630 | somatostatin receptor 2 |
| Cacng5 | 1.7 | 140723 | ENSMUST00000039071 | calcium channel, voltage-dependent, gamma subunit 5 |
| 1110067D22Rik | 1.7 | 216551 | AK041053 | RIKEN cDNA 1110067D22 gene |
| Azin1 | 1.7 | 54375 | NM_001102458 | antizyme inhibitor 1 |
| Cdh10 | 1.7 | 320873 | AK161927 | cadherin 10, type 2 (T2-cadherin) |
| Prei4 | 1.6 | 74182 | ENSMUST00000110137 | preimplantation protein 4 |
| Ddi2 | 1.6 | 68817 | AK078461 | DNA-damage inducible protein 2 |
| Cdkl2 | 1.6 | 53886 | NM_016912 | cyclin-dependent kinase-like 2 (CDC2-related kinase) |
| Nus1 | 1.6 | 52014 | AK160870 | nuclear undecaprenyl pyrophosphate synthase 1 homolog (S. cerevisiae) |
| Ppm1a | 1.6 | 19042 | AK009105 | protein phosphatase 1A (formerly 2C), magnesium-dependent, alpha isoform |
| C78339 | 1.6 | 97863 | AK090371 | expressed sequence C78339 |
| Efr3a | 1.6 | 76740 | AK044000 | EFR3 homolog A (S. cerevisiae) |
| Ier5 | 1.6 | 15939 | NM_010500 | immediate early response 5 |
| Pcmtd2 | 1.6 | 245867 | NM_153594 | protein-L-isoaspartate (D-aspartate) O-methyltransferase domain containing 2 |
| Pkd2 | 1.6 | 18764 | ENSMUST00000086831 | polycystic kidney disease 2 (autosomal dominant) |
| Nrip3 | 1.6 | 78593 | ENSMUST00000041460 | nuclear receptor interacting protein 3 |
| Mettl2 | 1.6 | 52686 | AK048040 | methyltransferase like 2B |
| Oxr1 | 1.6 | 170719 | NM_130885 | oxidation resistance 1 |
| 3110047M12Rik | 1.6 | 73184 | ENSMUST00000090095 | RIKEN cDNA 3110047M12 gene |
| Sfrs3 | 1.6 | 20383 | AK078891 | splicing factor, arginine/serine-rich 3 |
| Usp14 | 1.6 | 59025 | NM_001038589 | ubiquitin specific peptidase 14 (tRNA-guanine transglycosylase) |
| Abcb7 | 1.6 | 11306 | AK162299 | ATP-binding cassette, sub-family B (MDR/TAP), member 7 |
| St8sia4 | 1.6 | 20452 | ENSMUST00000043336 | ST8 alpha-N-acetyl-neuraminide alpha-2,8-sialyltransferase 4 |
| Pdik1l | 1.6 | 230809 | ENSMUST00000105877 | PDLIM1 interacting kinase 1 like |
| Tmem30a | 1.6 | 69981 | AK078136 | transmembrane protein 30A |
| Gng4 | 1.6 | 14706 | AK148452 | guanine nucleotide binding protein (G protein), gamma 4 |
| Psmc6 | 1.6 | 67089 | AK008996 | proteasome (prosome, macropain) 26S subunit, ATPase, 6 |
| Oxct1 | 1.6 | 67041 | AK154327 | 3-oxoacid CoA transferase 1 |
| Pvrl3 | 1.6 | 58998 | AK011949 | poliovirus receptor-related 3 |
| Dgkg | 1.6 | 110197 | NM_138650 | diacylglycerol kinase, gamma 90kDa |
| Fgd4 | 1.6 | 224014 | AK033429 | FYVE, RhoGEF and PH domain containing 4 |
| Tsr2 | 1.6 | 69499 | AK040910 | TSR2, 20S rRNA accumulation, homolog (S. cerevisiae) |
| Dpm1 | 1.6 | 13480 | AK004834 | dolichyl-phosphate mannosyltransferase polypeptide 1, catalytic subunit |
| Ppargc1a | 1.6 | 19017 | AK082685 | peroxisome proliferator-activated receptor gamma, coactivator 1 alpha |
| Sema3e | 1.6 | 20349 | AK032603 | sema domain, immunoglobulin domain (Ig), short basic domain, secreted, 3E |
| 2310045A20Rik | 1.6 | 231238 | AK009811 | RIKEN cDNA 2310045A20 gene |
| LOC100044162 | 1.6 | 100044162 | XM_001471667 | hypothetical protein LOC100044162 |
| Kcnd2 | 1.6 | 16508 | AK140148 | potassium voltage-gated channel, Shal-related subfamily, member 2 |
| Gpm6a | 1.6 | 234267 | AK003422 | glycoprotein M6A |
| Mtap4 | 1.6 | 17758 | AK044110 | microtubule-associated protein 4 |
| Dtna | 1.6 | 13527 | ENSMUST00000115832 | dystrobrevin, alpha |
| BC003331 | 1.6 | 226499 | ENSMUST00000094477 | cDNA sequence BC003331 |
| Usp45 | 1.6 | 77593 | AK013986 | ubiquitin specific peptidase 45 |
| Mef2a | 1.6 | 17258 | ENSMUST00000107476 | myocyte enhancer factor 2A |
| Homer2 | 1.6 | 26557 | AK003632 | homer homolog 2 (Drosophila) |
| Tspyl1 | 1.6 | 22110 | AK053812 | TSPY-like 1 |
| Ppp2r5c | 1.6 | 26931 | AK139212 | protein phosphatase 2, regulatory subunit B', gamma isoform |
| Mocs2 | 1.6 | 17434 | NM_001113375 | molybdenum cofactor synthesis 2 |
| Apc | 1.6 | 11789 | ENSMUST00000079362 | adenomatous polyposis coli |
| Riok3 | 1.6 | 66878 | AK008837 | RIO kinase 3 (yeast) |
| LOC100048863 | 1.6 | 100048863 | XM_001474062 | similar to adenomatosis polyposis coli |
| Snx30 | 1.6 | 209131 | AK047791 | sorting nexin family member 30 |
| Lmbr1 | 1.6 | 56873 | NM_020295 | limb region 1 homolog (mouse) |
| Cdh8 | 1.6 | 12564 | ENSMUST00000109459 | cadherin 8, type 2 |
| Arpp19 | 1.6 | 59046 | AK049760 | cyclic AMP phosphoprotein, 19 kD |
| Zc3h14 | 1.6 | 75553 | NM_029334 | zinc finger CCCH-type containing 14 |
| Slc35a5 | 1.6 | 74102 | NM_028756 | solute carrier family 35, member A5 |
| Map4k3 | 1.6 | 225028 | NM_001081357 | mitogen-activated protein kinase kinase kinase kinase 3 |
| LOC675560 | 1.6 | 675560 | XM_983140 | similar to mitogen-activated protein kinase kinase kinase kinase 3 |
| Blnk | 1.6 | 17060 | AK089704 | B-cell linker |
| Xiap | 1.6 | 11798 | AK078795 | X-linked inhibitor of apoptosis |
| Mecp2 | 1.6 | 17257 | NM_010788 | methyl CpG binding protein 2 (Rett syndrome) |
| 1700020I14Rik | 1.6 | 66602 | AK029148 | RIKEN cDNA 1700020I14 gene |
| Slc35a3 | 1.6 | 229782 | AK081236 | solute carrier family 35, member A3 |
| Eif4g3 | 1.6 | 230861 | AK133389 | eukaryotic translation initiation factor 4 gamma, 3 |
| Jhdm1d | 1.6 | 338523 | NM_001033430 | jumonji C domain containing histone demethylase 1 homolog D |
| LOC100047837 | 1.6 | 100047837 | XM_001478863 | similar to Myocyte enhancer factor 2A |
| Pgr | 1.6 | 18667 | AK137726 | progesterone receptor |
| Arpp21 | 1.6 | 74100 | AK034376 | cyclic AMP-regulated phosphoprotein, 21 kD |
| Sept7 | 1.6 | 235072 | ENSMUST00000060080 | septin 7 |
| Ank3 | 1.6 | 11735 | ENSMUST00000047061 | ankyrin 3, node of Ranvier (ankyrin G) |
| Bptf | 1.6 | 207165 | ENSMUST00000057892 | bromodomain PHD finger transcription factor |
| Pum2 | 1.6 | 80913 | AK147617 | pumilio homolog 2 (Drosophila) |
| Ankrd28 | 1.6 | 105522 | AK088541 | ankyrin repeat domain 28 |
| Lrrc6 | 1.6 | 54562 | ENSMUST00000023006 | leucine rich repeat containing 6 |
| Tmed7 | 1.6 | 66676 | AK077807 | transmembrane emp24 protein transport domain containing 7 |
| Arl5a | 1.6 | 75423 | AK147525 | ADP-ribosylation factor-like 5A |
| Slc4a10 | 1.6 | 94229 | AK047801 | solute carrier family 4, sodium bicarbonate transporter, member 10 |
| Trim2 | 1.6 | 80890 | ENSMUST00000107694 | tripartite motif-containing 2 |
| Usp10 | 1.6 | 22224 | AK165156 | ubiquitin specific peptidase 10 |
| Zcchc14 | 1.6 | 142682 | AK035149 | zinc finger, CCHC domain containing 14 |
| Tbcel | 1.6 | 272589 | AK046441 | tubulin folding cofactor E-like |
| Gpr83 | 1.6 | 14608 | AK030359 | G protein-coupled receptor 83 |
| Zfp280d | 1.6 | 235469 | NM_146224 | zinc finger protein 280D |
| Crk | 1.6 | 12928 | AK028488 | v-crk sarcoma virus CT10 oncogene homolog (avian) |
| Matn2 | 1.6 | 17181 | AK138211 | matrilin 2 |
| Mobkl3 | 1.6 | 19070 | AK034840 | MOB1, Mps One Binder kinase activator-like 3 (yeast) |
| Atp1a2 | 1.6 | 98660 | AK147331 | ATPase, Na+/K+ transporting, alpha 2 (+) polypeptide |
| 4930431H11Rik | 1.6 | 73785 | AK015268 | RIKEN cDNA 4930431H11 gene |
| Synj2bp | 1.6 | 24071 | AK078422 | synaptojanin 2 binding protein |
| Edil3 | 1.6 | 13612 | AK081003 | EGF-like repeats and discoidin I-like domains 3 |
| Gmfb | 1.6 | 63985 | AK142014 | glia maturation factor, beta |
| 9030612M13Rik | 1.6 | 208292 | AK052844 | RIKEN cDNA 9030612M13 gene |
| 2310051E17Rik | 1.6 | 70273 | XM_001479552 | RIKEN cDNA 2310051E17 gene |
| Ddx3x | 1.6 | 13205 | AK140990 | DEAD (Asp-Glu-Ala-Asp) box polypeptide 3, X-linked |
| 3110035E14Rik | 1.5 | 76982 | NM_178399 | RIKEN cDNA 3110035E14 gene |
| Fmnl2 | 1.5 | 71409 | AK080318 | formin-like 2 |
| Unc13b | 1.5 | 22249 | NM_001081413 | unc-13 homolog B (C. elegans) |
| Atp2a2 | 1.5 | 11938 | ENSMUST00000031423 | ATPase, Ca++ transporting, cardiac muscle, slow twitch 2 |
| Tmed5 | 1.5 | 73130 | AK033463 | transmembrane emp24 protein transport domain containing 5 |
| LOC100046567 | 1.5 | 100046567 | XM_001476494 | similar to Transmembrane emp24 protein transport domain containing 5 |
| Nup93 | 1.5 | 71805 | NM_172410 | nucleoporin 93kDa |
| Neto2 | 1.5 | 74513 | ENSMUST00000047344 | neuropilin (NRP) and tolloid (TLL)-like 2 |
| LOC100039128 | 1.5 | 100039128 | XM_001472320 | similar to nucleoporin 93 |
| Aph1b | 1.5 | 208117 | AK154847 | anterior pharynx defective 1 homolog B (C. elegans) |
| Suz12 | 1.5 | 52615 | AK163993 | suppressor of zeste 12 homolog (Drosophila) |
| Xpo1 | 1.5 | 103573 | ENSMUST00000102870 | exportin 1 (CRM1 homolog, yeast) |
| Sostdc1 | 1.5 | 66042 | AK081954 | sclerostin domain containing 1 |
| Ctage5 | 1.5 | 217615 | NM_146034 | CTAGE family, member 5 |
| AA987161 | 1.5 | 380856 | XM_906771 | expressed sequence AA987161 |
| Ophn1 | 1.5 | 94190 | ENSMUST00000113815 | oligophrenin 1 |
| Enah | 1.5 | 13800 | AK076131 | enabled homolog (Drosophila) |
| MFSD6 | 1.5 | 98682 | NM_133829 | major facilitator superfamily domain containing 6 |
| Sfrs6 | 1.5 | 67996 | NM_026499 | splicing factor, arginine/serine-rich 6 |
| Phf20 | 1.5 | 228829 | NM_172674 | PHD finger protein 20 |
| Abcd3 | 1.5 | 19299 | AK004660 | ATP-binding cassette, sub-family D (ALD), member 3 |
| Larp2 | 1.5 | 214048 | AK160155 | La ribonucleoprotein domain family, member 2 |
| Elavl4 | 1.5 | 15572 | AK014133 | ELAV (embryonic lethal, abnormal vision, Drosophila)-like 4 |
| 1110054O05Rik | 1.5 | 66209 | AK012023 | RIKEN cDNA 1110054O05 gene |
| Mllt3 | 1.5 | 70122 | ENSMUST00000097995 | myeloid/lymphoid or mixed-lineage leukemia; translocated to, 3 |
| Uba3 | 1.5 | 22200 | ENSMUST00000032142 | ubiquitin-like modifier activating enzyme 3 |
| Aebp2 | 1.5 | 11569 | NM_001005605 | AE binding protein 2 |
| Casd1 | 1.5 | 213819 | NM_145398 | CAS1 domain containing 1 |
| LOC100045658 | 1.5 | 100045658 | XM_001474684 | similar to O-acetyltransferase |
| Atp2c1 | 1.5 | 235574 | ENSMUST00000112557 | ATPase, Ca++ transporting, type 2C, member 1 |
| LOC100045884 | 1.5 | 100045884 | XM_001475215 | hypothetical protein LOC100045884 |
| Mtmr6 | 1.5 | 219135 | AK076218 | myotubularin related protein 6 |
| Armcx5 | 1.5 | 494468 | AK133950 | armadillo repeat containing, X-linked 5 |
| D1Ertd53e | 1.5 | 213056 | AK048435 | family with sequence similarity 126, member B |
| Abi1 | 1.5 | 11308 | ENSMUST00000114544 | abl-interactor 1 |
| Ermn | 1.5 | 77767 | AK137305 | ermin, ERM-like protein |
| Eif4e | 1.5 | 13684 | NM_007917 | eukaryotic translation initiation factor 4E |
| Hiat1 | 1.5 | 15247 | AK004779 | hippocampus abundant transcript 1 |
| LOC630527 | 1.5 | 630527 | XM_925747 | hypothetical LOC630527 |
| Prdm2 | 1.5 | 110593 | ENSMUST00000084200 | PR domain containing 2, with ZNF domain |
| Impad1 | 1.5 | 242291 | AK041202 | inositol monophosphatase domain containing 1 |
| Sgcb | 1.5 | 24051 | AK014381 | sarcoglycan, beta (43kDa dystrophin-associated glycoprotein) |
| Tgfa | 1.5 | 21802 | AK162680 | transforming growth factor, alpha |
| Slco1a4 | 1.5 | 28250 | ENSMUST00000032364 | solute carrier organic anion transporter family, member 1A2 |
| Wwc2 | 1.5 | 52357 | AK028956 | WW and C2 domain containing 2 |
| Myo5a | 1.5 | 17918 | ENSMUST00000113486 | myosin VA (heavy chain 12, myoxin) |
| Sorl1 | 1.5 | 20660 | NM_011436 | sortilin-related receptor, L(DLR class) A repeats-containing |
| Ugp2 | 1.5 | 216558 | ENSMUST00000060895 | UDP-glucose pyrophosphorylase 2 |
| Vps54 | 1.5 | 245944 | AK137211 | vacuolar protein sorting 54 homolog (S. cerevisiae) |
| Homer1 | 1.5 | 26556 | NM_152134 | homer homolog 1 (Drosophila) |
| Rcan2 | 1.5 | 53901 | NM_030598 | regulator of calcineurin 2 |
| Utx | 1.5 | 22289 | NM_009483 | ubiquitously transcribed tetratricopeptide repeat, X chromosome |
| Pik3ca | 1.5 | 18706 | ENSMUST00000108242 | phosphoinositide-3-kinase, catalytic, alpha polypeptide |
| Elavl2 | 1.5 | 15569 | ENSMUST00000107124 | ELAV (embryonic lethal, abnormal vision, Drosophila)-like 2 |
| Tgoln1 | 1.5 | 22134 | NM_009443 | trans-golgi network protein 2 |
| Hnrnpa2b1 | 1.5 | 53379 | NM_182650 | heterogeneous nuclear ribonucleoprotein A2/B1 |
| B630005N14Rik | 1.5 | 101148 | AK135664 | RIKEN cDNA B630005N14 gene |
| Slc6a15 | 1.5 | 103098 | AK036136 | solute carrier family 6 (neutral amino acid transporter), member 15 |
| Ctsb | 1.5 | 13030 | NM_007798 | cathepsin B |
| Brwd1 | 1.5 | 93871 | AK027961 | bromodomain and WD repeat domain containing 1 |
| Ets2 | 1.5 | 23872 | AK145852 | v-ets erythroblastosis virus E26 oncogene homolog 2 (avian) |
| Tomm70a | 1.5 | 28185 | AK028543 | translocase of outer mitochondrial membrane 70 homolog A |
| Zdhhc23 | 1.5 | 332175 | AK137472 | zinc finger, DHHC-type containing 23 |
| B4galt6 | 1.5 | 56386 | AK144997 | UDP-Gal:betaGlcNAc beta 1,4- galactosyltransferase, polypeptide 6 |
| LOC675709 | 1.5 | 675709 | XM_984471 |  |
| Stx3 | 1.5 | 20908 | ENSMUST00000075304 | syntaxin 3 |
| Vldlr | 1.5 | 22359 | ENSMUST00000047645 | very low density lipoprotein receptor |
| Tmx4 | 1.5 | 52837 | AK040779 | thioredoxin-related transmembrane protein 4 |
| Arhgap21 | 1.5 | 71435 | NM_001128084 | Rho GTPase activating protein 21 |
| Strbp | 1.5 | 319517 | AK045193 | spermatid perinuclear RNA binding protein |
| LOC100045887 | 1.5 | 100045887 | XM_001475106 | similar to PTB-associated splicing factor |
| Prkcb1 | 1.5 | 18751 | AK051862 | protein kinase C, beta |
| Rspry1 | 1.5 | 67610 | AK076799 | ring finger and SPRY domain containing 1 |
| Tlr3 | 1.5 | 142980 | AK166177 | toll-like receptor 3 |
| Mobp | 1.5 | 17433 | AK083103 | myelin-associated oligodendrocyte basic protein |
| Fam63b | 1.5 | 235461 | AK077470 | family with sequence similarity 63, member B |
| Map4k5 | 1.5 | 399510 | AK019468 | mitogen-activated protein kinase kinase kinase kinase 5 |
| Gtpbp4 | 1.5 | 69237 | AK166711 | GTP binding protein 4 |
| Rasa1 | 1.5 | 218397 | ENSMUST00000022034 | RAS p21 protein activator (GTPase activating protein) 1 |
| Rb1 | 1.5 | 19645 | AK146467 | retinoblastoma 1 |
| Slitrk1 | 1.5 | 76965 | AK014285 | SLIT and NTRK-like family, member 1 |
| Mycbp2 | 1.5 | 105689 | ENSMUST00000039876 | MYC binding protein 2 |
| Cep97 | 1.5 | 74201 | AK053640 | centrosomal protein 97kDa |
| Pmaip1 | 1.5 | 58801 | AK043856 | phorbol-12-myristate-13-acetate-induced protein 1 |
| Fam59a | 1.5 | 381126 | AK162177 | family with sequence similarity 59, member A |
| Rbm4b | 1.5 | 66704 | AK028347 | RNA binding motif protein 4B |
| Il33 | 1.5 | 77125 | NM_133775 | interleukin 33 |
| Stag2 | 1.5 | 20843 | AK088799 | stromal antigen 2 |
| Cstf2 | 1.5 | 108062 | AK088260 | cleavage stimulation factor, 3' pre-RNA, subunit 2, 64kDa |
| Egfl7 | -1.5 | 353156 | NM_198725 | EGF-like-domain, multiple 7 |
| Psmc5 | -1.5 | 19184 | AK010505 | proteasome (prosome, macropain) 26S subunit, ATPase, 5 |
| Serpinf1 | -1.5 | 20317 | NM_011340 | serpin peptidase inhibitor, clade F, member 1 |
| Prdx5 | -1.5 | 54683 | AK002383 | peroxiredoxin 5 |
| Tbcb | -1.5 | 66411 | AK002316 | tubulin folding cofactor B |
| Exosc6 | -1.5 | 72544 | AK088532 | exosome component 6 |
| Hcfc1r1 | -1.5 | 353502 | AK003865 | host cell factor C1 regulator 1 (XPO1 dependent) |
| Krtcap2 | -1.5 | 66059 | NM_025327 | keratinocyte associated protein 2 |
| 2310028O11Rik | -1.5 | 433771 | AK159039 | RIKEN cDNA 2310028O11 gene |
| Calm3 | -1.5 | 12315 | AK151610 | calmodulin 3 (phosphorylase kinase, delta) |
| Reep5 | -1.5 | 13476 | AK010507 | receptor accessory protein 5 |
| Gm6563 | -1.5 | 625193 | ENSMUST00000056396 | predicted gene 6563 |
| Npdc1 | -1.5 | 18146 | NM_008721 | neural proliferation, differentiation and control, 1 |
| Apoa1bp | -1.5 | 246703 | NM_144897 | apolipoprotein A-I binding protein |
| Arhgdia | -1.5 | 192662 | NM_133796 | Rho GDP dissociation inhibitor (GDI) alpha |
| Rbm26 | -1.5 | 74213 | AK049083 | RNA binding motif protein 26 |
| Pcsk1n | -1.6 | 30052 | AK076137 | proprotein convertase subtilisin/kexin type 1 inhibitor |
| Nrgn | -1.6 | 64011 | NM_022029 | neurogranin (protein kinase C substrate, RC3) |
| Car4 | -1.6 | 12351 | ENSMUST00000108076 | carbonic anhydrase IV |
| H2afv | -1.6 | 77605 | AK021267 | H2A histone family, member V |
| Rpl14 | -1.6 | 67115 | AK141887 | ribosomal protein L14 |
| Cdk4 | -1.6 | 12567 | AK019380 | cyclin-dependent kinase 4 |
| Gtf3c6 | -1.6 | 67371 | NM_026113 | general transcription factor IIIC, polypeptide 6, alpha 35kDa |
| LOC640611 | -1.6 | 640611 | XM_917659 | similar to Cell division protein kinase 4 |
| Pdxp | -1.6 | 57028 | AK043228 | pyridoxal (pyridoxine, vitamin B6) phosphatase |
| Prr13 | -1.6 | 66151 | AK002430 | proline rich 13 |
| Fam108a | -1.6 | 216169 | AK170785 | family with sequence similarity 108, member A |
| Lin7b | -1.6 | 22342 | AK019299 | lin-7 homolog B (C. elegans) |
| Abhd8 | -1.6 | 64296 | NM_022419 | abhydrolase domain containing 8 |
| Bola2 | -1.6 | 66162 | AK140279 | bolA homolog 2 (E. coli) |
| Tfpt | -1.6 | 69714 | ENSMUST00000108641 | TCF3 (E2A) fusion partner (in childhood Leukemia) |
| Tubb3 | -1.6 | 22152 | AK012528 | tubulin, beta 3 |
| Rab24 | -1.6 | 19336 | ENSMUST00000035242 | RAB24, member RAS oncogene family |
| Rbm42 | -1.6 | 68035 | ENSMUST00000108141 | RNA binding motif protein 42 |
| l7Rn6 | -1.7 | 67669 | ENSMUST00000075010 | lethal, Chr 7, Rinchik 6 |
| Ehd1 | -1.7 | 13660 | NM_010119 | EH-domain containing 1 |
| Ndufc2 | -1.7 | 68197 | NM_024220 | NADH dehydrogenase (ubiquinone) 1, subcomplex unknown, 2, 14.5kDa |
| LOC635087 | -1.7 | 635087 | XM_910031 | similar to NADH dehydrogenase 1, subcomplex unknown, 2 |
| LOC675851 | -1.7 | 675851 | XM_985615 | similar to NADH dehydrogenase 1, subcomplex unknown, 2 |
| Dcxr | -1.7 | 67880 | AK002803 | dicarbonyl/L-xylulose reductase |
| Arpc4 | -1.7 | 68089 | AK159410 | actin related protein 2/3 complex, subunit 4, 20kDa |
| Dad1 | -1.7 | 13135 | AK002446 | defender against cell death 1 |
| Dhcr7 | -1.7 | 13360 | AK146811 | 7-dehydrocholesterol reductase |
| Sncb | -1.7 | 104069 | NM_033610 | synuclein, beta |
| Hspbp1 | -1.7 | 66245 | NM_024172 | HSPA (heat shock 70kDa) binding protein, cytoplasmic cochaperone 1 |
| Banf1 | -1.7 | 23825 | NM_011793 | barrier to autointegration factor 1 |
| Ramp2 | -1.7 | 54409 | NM_019444 | receptor (G protein-coupled) activity modifying protein 2 |
| Ndufaf2 | -1.7 | 75597 | AK007894 | NADH dehydrogenase 1 alpha subcomplex, assembly factor 2 |
| Mrpl52 | -1.7 | 68836 | AK004194 | mitochondrial ribosomal protein L52 |
| Ttc9b | -1.7 | 73032 | AK038864 | tetratricopeptide repeat domain 9B |
| Syt17 | -1.8 | 110058 | AK144560 | synaptotagmin XVII |
| Sst | -1.8 | 20604 | AK003014 | somatostatin |
| Ccr5 | -1.8 | 12774 | NM_009917 | chemokine (C-C motif) receptor 5 |
| B230220B15Rik | -1.8 | 320723 | AK080810 | RIKEN cDNA B230220B15 gene |
| Fkbp2 | -1.8 | 14227 | AK144284 | FK506 binding protein 2, 13kDa |
| Sepw1 | -2.0 | 20364 | AK002870 | selenoprotein W, 1 |

**Table S2.** Selected significantly (p<0.05) enriched functional annotations associated with the 274 genes regulated in the CA3 region of UBB^+1^ tg mice compared to control animals. Shown are human gene symbols as provided by Ingenuity. Downregulation is indicated by an arrow, otherwise genes were upregulated.


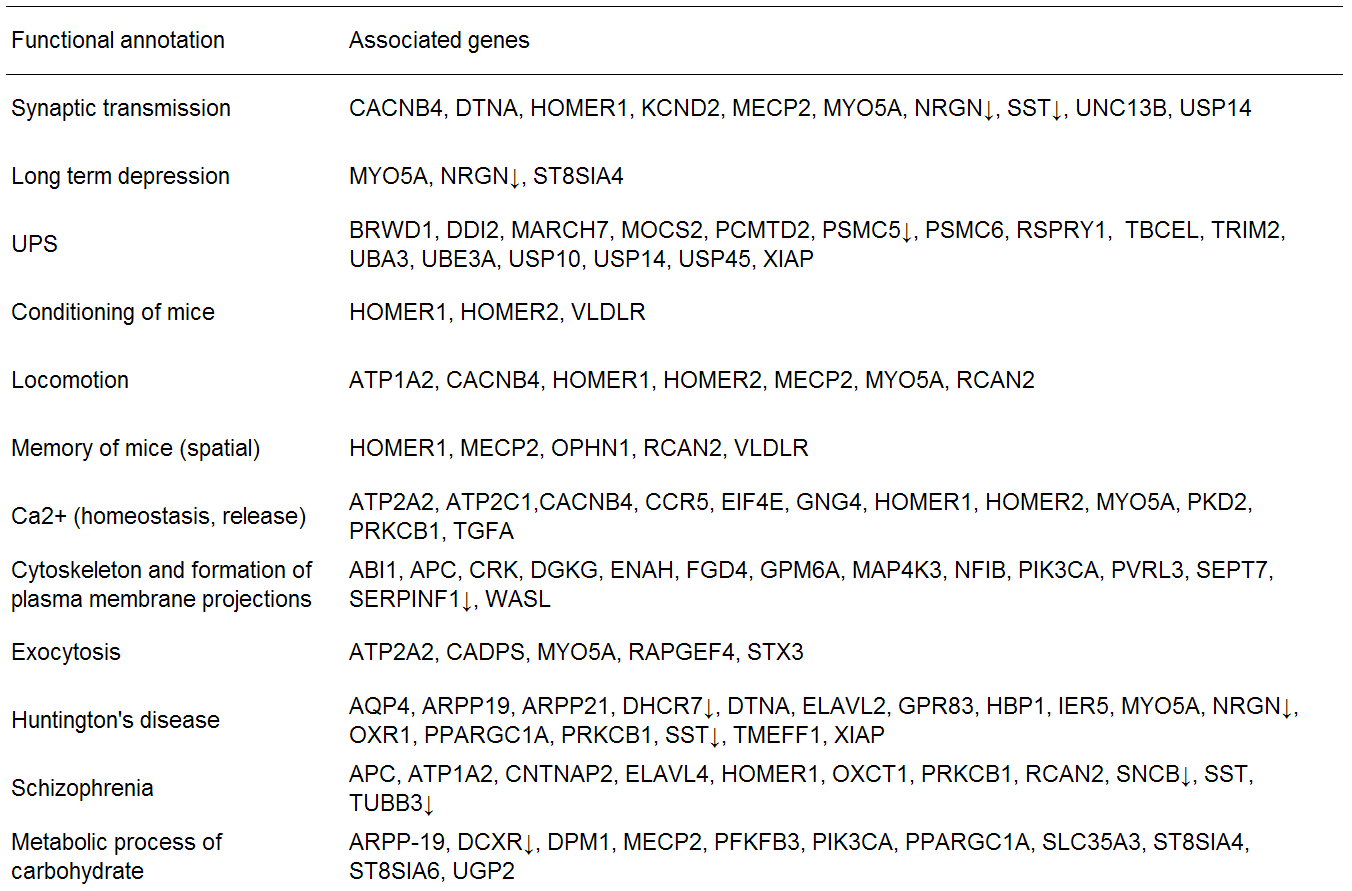


**Table S3.** UPS-related genes regulated in the CA3 region of UBB^+1^ tg mice: biological functions and associated diseases

| **Gene symbol** | **Entrez ID** | **UPS function** | **Fold-change (linear)** | **Gene name** | **Related disorder** | **Biological function** |
| --- | --- | --- | --- | --- | --- | --- |
| Ube3a | 22215 | Ligase simple; HECT | 1.7 | ubiquitin protein ligase E3A | Angelman Syndrom, Autism | • Mice with maternally inherited targeted null mutations exhibit reduced brain weight, impaired motor function, inducible seizures, learning deficits, abnormal hippocampal electroencephalographic recordings, and severely impaired long-term potentiation • May regulate PI3K pathway |
| Usp14 | 59025 | Protease UBP | 1.6 | ubiquitin specific peptidase 14 (tRNA-guanine transglycosylase) | Ataxia, synaptic dysfunction | • Homozygotes for a hypomorphic mutation develop severe tremors by 3 weeks of age, followed by hindlimb paralysis and premature death. An underdeveloped corpus callosum, hippocampus, dentate gyrus and forebrain structures, and notable defects in synaptic transmission in both the CNS and PNS are seen. Loss of Usp14 results in decreased levels of ubiquitin • Ataxia (axj) mutation leads to reduced Usp14 expression and neurological disease |
| Brwd1 | 93871 | Ligase complex;DDB1-adapters | 1.5 | bromodomain and WD repeat domain containing 1 | Down syndrome | • Required for normal spermiogenesis and the oocyte-embryo transition.J3 • Putative transcriptional regulator that may act on chromatin through interactions with the Brg1-dependent SWI/SNF chromatin-remodeling • Maps to Down syndrome critical region |
| Usp10 | 22224 | Protease UBP | 1.6 | ubiquitin specific peptidase 10 | Glioblastoma | • Regulates p53 localization and stability by deubiquitinating p53 • Regulates postendocytic sorting of Cftr and other proteins • Associated with poor survival in glioblastoma multiforme |
| Mocs2 | 17434 | Ub-like/fold; Ub-like | 1.6 | molybdenum cofactor synthesis 2 | Neurodegeneration | • Human Moco deficiency is a hereditary metabolic disorder characterized by severe neurodegeneration resulting in early childhood death, which results primarily from the deficiency of sulfite oxidase |
| Psmc5 | 19184 | Proteasome | -1.5 | proteasome (prosome, macropain) 26S subunit, ATPase, 5 | AD, Machado–Joseph disease | • Degrades ataxin-3 in Machado–Joseph disease, which is a neurodegenerative disorder caused by an expansion of the polyglutamine tract of ataxin-3 • Regulated by long-term depression and by phosphorylation (via PKA) • Inhibition of Psmc5 upregulates Myo18b |
| Xiap | 11798 | Ligase simple; Ring | 1.6 | X-linked inhibitor of apoptosis | Neurodegeneration | • S-nitrosylation of XIAP compromises neuronal survival in Parkinson's disease • Postsynaptic function in learning and memory • Expression of Xiap in mature Purkinje cells and in retinal bipolar cells in transgenic mice induces neurodegeneration • Inhibition/activation of apoptosis |
| Uba3 | 22200 | E1 | 1.5 | ubiquitin-like modifier activating enzyme 3 | Neurological disorders | • APPBP1-UBA3 functions as heterodimeric E1 enzyme for NEDD8 activation. Nedd8 regulates ubiquitin E3 ligases. • Homozygous null mutants die at the peri-implantation stage and exhibit selective apoptosis of the inner cell mass |
| March7 | 57438 | Ligase simple; Ring | 1.7 | membrane-associated ring finger (C3HC4) 7 | Neural development | • Homozygous null mice show premature neural degeneration and defective development of the corpus callosum. Both T cell proliferation and T cell-derived leukaemia inhibitory factor are increased. • Stabilized by deubiquitination (by USP7 and USP9X) |
| Pcmtd2 | 245867 | Ligase complex; SOCS-box | 1.6 | protein-L-isoaspartate (D-aspartate) O-methyltransferase domain containing 2 | Mental retardation | • Unknown function • Loss of Pcmtd2 and Myt1 results in mental retardation |
| Trim2 | 80890 | Ligase simple; Ring | 1.6 | tripartite motif-containing 2 | Neurodegeneration | • Deficiency in ubiquitin ligase TRIM2 causes accumulation of eurofilament light chain and neurodegeneration • Neuronal differentiation • May bind to myosin V • Mice homozygous for a gene trapped allele exhibit tremors, ataxia and seizures associated with neurodegeneration of Purkinje cells, deep cerebellar nuclei and retinal ganglion cells. |
| Usp45 | 77593 | Protease UBP | 1.6 | ubiquitin specific peptidase 45 |  | • Unknown function |
| Ddi2 | 68817 | Ub-like/fold; Ub-like | 1.6 | DNA-damage inducible protein 2 |  | • Unknown function • Human homologue unclear (Rsc1a1?) |
| Psmc6 | 67089 | Proteasome | 1.6 | proteasome (prosome, macropain) 26S subunit, ATPase, 6 |  | • Overexpression of Psmc6 or Psmc5 facilitated aggregation of mutant huntingtin and ataxin-3 without affecting proteasomal degradation |
| Rspry1 | 67610 | Ligase simple; Ring | 1.5 | ring finger and SPRY domain containing 1 |  | • Unknown function |
| Tbcel | 272589 | Ub-like/fold; Ub-like | 1.6 | tubulin folding cofactor E-like |  | • Tubulin-destabilizing protein related to the chaperone cofactor E |

**Table S4. Represented are genes, which were tested for mRNA expression by qPCR. Bold genes are statistically significant different in UBB^+1^ tg mice compared to age matched controls (p<0.05)**

| Group | Subgroup | Gene (abbreviation) | Gene (full name) | Function |
| --- | --- | --- | --- | --- |
| Synaptic functioning | Presynaptic function | **SNAP25** | **Synaptosomal-associated protein 25** | **t-SNARE involved in the molecular regulation of neurotransmitter release.** |
|  |  | SYP | Synaptophysin | Targetting vesicles to the presynaptic membrane |
|  |  | RIMBP2 | RIMS-binding protein 2 | Involved in synaptic transmission |
|  |  | STX1A | **Syntaxin-1A** | Invlolved in docking of synaptic vesicles at presynaptic synaptic membrane |
|  | Postsynaptic function | GLUR1 | Ionotropic glutamate receptor 1 | Binding of L-glutamate leads to conformational change and opens its cation channel |
|  |  | **GLUR2** | **Ionotropic glutamate receptor 2** | **Binding of L-glutamate leads to conformational change and opens its cation channel** |
|  |  | GLUR3 | Ionotropic glutamate receptor 3 | Binding of L-glutamate leads to conformational change and opens its cation channel |
|  |  | GLUR4 | Ionotropic glutamate receptor 4 | Binding of L-glutamate leads to conformational change and opens its cation channel |
|  |  | GRIN1 | Glutamate [NMDA] receptor subunit zeta-1 | NMDA receptor subtype of glutamate-gated ion channels with high calcium permeability |
|  |  | **GRIN2A** | **Glutamate [NMDA] receptor subunit epsilon-1** | **NMDA receptor subtype of glutamate-gated ion channels with high calcium permeability** |
|  |  | DLG4 | Disks large homolog 4 | Binds to and modulates NMDA receptor subunits |
|  |  | **CAMK2A** | **Calcium/calmodulin-dependent protein kinase**  **type II subunit alpha** | **Kinase important for long-term potentiation and neurotransmitter release. It may regulate NMDA receptor dependent potentiation.** |
|  |  | GABRA5 | Gamma-aminobutyric acid  receptor subunit alpha-5 | Receptor for GABA which opens a chloride channel and inhibits the post-synaptic neuron |
|  |  | **GABRB2** | **Gamma-aminobutyric acid type B receptor subunit 2** | **Receptor for GABA which opens a chloride channel and inhibits the post-synaptic neuron** |
| AD-related |  | **APP** | **Amyloid precursor protein** | **Precursor for amyloid-β 40 and 42 peptides** |
|  |  | BACE1 | **Beta-secretase 1** | Catalytic subunit of β-secretase complex. Proteolytic processing of APP for the amyloidogenic pathway |
|  |  | PS1 | Presenilin 1 | Catalytic subunit of γ-secretase complex. Proteolytic processing for the amyloidogenic pathway |
| Protein quality control | UBB^+1^-related | UCH-L3 | Ubiquitin C-terminal hydrolase L3 | Hydrolase which is able to remove the C-teminal extension of UBB^+1^ |
|  |  | E2-25K | Ubiquitin-conjugating enzyme E2-25 kDa | E2-conjugating enzyme which is able to ubiquitinate UBB^+1^ |
|  |  | TRIP12 | Thyroid receptor-interacting protein 12 | E3-ligase which is able to ubiquitinated UBB^+1^ |
|  | UPS-related | NEDD8 | Neural precursor cell expressed  developmentally down-regulated protein 8 | Ubiquitin like protein. Plays important role in cell cycle control and embryogenesis |
|  |  | ADRM1 | Proteasomal ubiquitin receptor ADRM1 | Proteasome ubiquitin receptor which also requites UCH-L5 |
|  |  | UCH-L1 | Ubiquitin C-terminal hydrolase L1 | Processing ubiquitin by removing (small) C-teminal extensions  Contrary to UCH-L3, does not process UBB^+1^ |
|  |  | UCH-L5 | Ubiquitin C-terminal hydrolase L5 | Processing ubiquitin by removing (small) C-teminal extensions  Contrary to UCH-L3, does not process UBB^+1^. Proteasome bound. |
|  |  | UBQLN1 | Ubiquilin 1 | Regulates trafficking of membrane bound proteins to the membranes. Involved in translocation of APP and PS1 |
|  |  | GRP78 | 78 kDa glucose-regulated protein | Chaparone which is important for the unfolded protein response (UPR) |
|  |  | RS27A | Ubiquitin-40S ribosomal protein S27a | Ribosomal protein ubiquitin fusion. |
|  | Autophagy-related | **MAP1LC3A** | Autophagy-related protein LC3 A | Ubiquitin-like protein involved in formation of autophagosomal vacuoles |
|  |  | ATG12 | Autophagy-related protein 12 | Ubiquitin-like protein essential for autophagy |
| Miscella-neous |  | GRN | Granulins | Cytokine-like activity. Mutations in the GRN gene can cause frontotemporal dementia |
|  |  | GFAP | **Glial fibrillary acidic protein** | Astrocyte specific protein often used as a marker for astroglyosis |
|  |  | DAB1 | **Disabled homolog 1** | Pivotal regulator of the Reelin signalling |
|  |  | SIRT1 | **NAD-dependent deacetylase sirtuin-1** | Improves insulin sensitivity and reduces processing of APP via the amyloidogenic pathway |
|  |  | **YWHAZ** | **14-3-3 protein zeta/delta** | **Adapter protein also involved in many processes. Can serve as a heat shock protein and can solubilise aggregated proteins** |

**Table S5.** Sequences of the oligos used for qPCR

| Gene | Full name | Primer sense | Primer antisense |
| --- | --- | --- | --- |
| 14-3-3 zeta | 14-3-3 protein zeta | TCGTACAAAGACAGCACGC | AAGGCCGGTTAATTTTCCCC |
| ADRM1 | Proteasomal ubiquitin receptor ADRM1 | AGAACACATTAACCTCGCCC | CGAACTGGCACATGAGAGG |
| APP | Amyloid beta A4 protein | GATTCAGGATTTGAAGTCCGC | GCCCACCATGAGTCCG |
| ATG12 | Ubiquitin-like protein ATG12 | ATGTGAATCAGTCCTTTGCCC | GACCAGTTTACCATCACTGCC |
| ATG8 | Gamma-aminobutyric acid receptor-associated protein-like 1 | CCTGGATAAGACCAAGTTTCTGG | GTTGACCAGCAGGAAGAAGG |
| BACE1 | Beta-secretase 1 | GCTACTATGTGGAGATGACCG | TGCAAAGTTACTACTGCCCG |
| CaMKIIa | Calcium/calmodulin-dependent protein kinase type II subunit alpha | TACCATCACCTGCACCCG | CACCTTCACACACCTGCG |
| DAB1 | Disabled homolog 1 | CTTTGATGAGAAGACGGGGG | AAACGTATCCGAAAGCCCG |
| DLG4 | Disks large homolog 4 | CCAACGATGATCTTCTCTCCG | CCGTCTATCTCATATTCCCGC |
| E2-25K | Ubiquitin-conjugating enzyme E2-25 kDa | CAGACACACCGTATGAAGGC | ATAAACCGGACCTTAGGGGG |
| GABRA5 | Gamma-aminobutyric acid receptor subunit alpha-5 | GGATGATGGCACACTTCTCTAC | ACTGCCAAATTTCAGGGGAC |
| GABRB2 | Gamma-aminobutyric acid receptor subunit beta-2 | GCTGCTAATGCCAACAATGAG | ATTACTGCTTCGGATGTGGC |
| GAPDH | Glyceraldehyde-3-phosphate dehydrogenase | AAAAGGGTCATCATCTCCGC | ATTTCTCGTGGTTCACACCC |
| GFAP | Glial fibrillary acidic protein | ATTCGCACTCAATACGAGGC | GAGGTCTGCAAACTTAGACCG |
| GLUR1 | Glutamate receptor 1 | TGGACTGTGAATCAGAACGC | AGTCAATGTCCATGAAGCCC |
| GLUR2 | Glutamate receptor 2 | ATCGACAATTTGGAGGTAGCC | CCAAAAATCGCATAGACGCC |
| GLUR3 | Glutamate receptor 3 | CAAATGGTGGTACGATAAGGGG | CCGACAAGTATATAGAACACGCC |
| GLUR4 | Glutamate receptor 4 | GAGACTGTACGCCAAAACCC | AGAGGCATTGAAGATGATGGC |
| GRIN1 | Glutamate [NMDA] receptor subunit zeta-1 | TGTCAGTTCATGTGGTGGC | ACCTTAAATCGGCCAAAGGG |
| GRIN2A | Glutamate [NMDA] receptor subunit epsilon-1 | TCTCCTACATTCCCGAGGC | GACAAGTCCTTGCCATCCC |
| GRN | Progranulin | CACACCCTACTAAAGAAGTTCCC | CACAGCAGGTAGAATCATCGG |
| GRP78 | 78 kDa glucose-regulated protein | CACTTGGAATGACCCTTCGG | GCAAATGTCTTGGTTTGCCC |
| HPRT1 | Hypoxanthine-guanine phosphoribosyltransferase | AAGTTTGTTGTTGGATATGCCC | ACTTGCGCTCATCTTAGGC |
| NEDD8 | NEDD8 | AGATTGAGATAGACATCGAACCC | CCTTCTTTTTCTTCCACACGC |
| PS1 | Presenilin 1 | GAATATGGCTGAAGGAGACCC | GCCACCATCATCGTTCCC |
| RIMBP | RIMS-binding protein | GGGACATGGATGAAGATGGG | ATCCACAAAGTTGGAGGGC |
| RS27a | Ubiquitin-40S ribosomal protein S27a | CAGAGGCTGATCTTTGCTGG | GTCTCAACACCAGATGAAGGG |
| SNAP25 | Synaptosomal-associated protein 25 | GGGCAATGAGATTGACACCC | CTTTGTTGCACGTTGGTTGG |
| SRT1 | NAD-dependent deacetylase sirtuin-1 | AGTTCTGACTGGAGCTGGG | CAAGGCGAGCATAGATACCG |
| STX1 | Syntaxin-1A | AGGAGGAAGGTCTGAACCG | ACAAACTTTCGGGACAGCG |
| SYP | Synaptophysin | GGTGAATCAGCTGGTGGC | TAGCAAAGGCGAAGATGGC |
| TRIP12 | Probable E3 ubiquitin-protein ligase TRIP12 | ATTTGAATCGGTCTTCCCGC | GCATCCCAAGTGTCTGCTTTA |
| UBQLN1 | Ubiquilin-1 | TACACCGATATCCAGGAGCC | TTCGAGAAGGCTGAGTCCC |
| UCH-L1 | Ubiquitin carboxyl-terminal hydrolase isozyme L1 | GAAGCCGATGGAGATTAACCC | GAGTCTCCTCCTCCAGCC |
| UCH-L3 | Ubiquitin carboxyl-terminal hydrolase isozyme L3 | CGGCTGTCAGAGCTGGAG | ATGCAGGCCTAACTGCTTGA |
| UCH-L5 | Ubiquitin carboxyl-terminal hydrolase isozyme L5 | ACTAGACGGGTTAAGAGAGGG | CTCTATTACTGGCCTCACTGC |

**Supplementary abbreviations**

bw body weight (g)

mean_f mean of all respiratory rates (1/min)

f respiratory rate (1/min)

TV tidal volume (ml)

sTV specific tidal volume (µl/g)

MV minute ventilation (ml/min)

sMV specific ventilation (ml/min/g)

Ti inspiratory time (ms)

Te expiratory time (ms)

Ti/TT relative duration of inspiration

PIF peak inspiratory flow rate (ml/s)

PEF peak expiratory flow rate (ml/s)

MIF mean inspiratory flow rate (ml/s)

MEF mean expiratory flow rate (ml/s)

**Supplementary references**

1. Bass J, Takahashi JS (2010) Circadian integration of metabolism and energetics. Science 330:1349-1354

2. Bode M, Irmler M, Friedenberger M, May C, Jung K, Stephan C, Meyer HE, Lach C, Hillert R, Krusche A, et al. (2008) Interlocking transcriptomics, proteomics and toponomics technologies for brain tissue analysis in murine hippocampus. Proteomics 8:1170-1178

3. Burns JM, Mayo MS, Anderson HS, Smith HJ, Donnelly JE (2008) Cardiorespiratory fitness in early-stage Alzheimer disease. Alz. Dis. Assoc. Dis. 22:39-46

4. Dennissen FJ, Kholod N, Hermes DJ, Kemmerling N, Steinbusch HW, Dantuma NP, van Leeuwen FW (2011) Mutant ubiquitin (UBB(+1)) associated with neurodegenerative disorders is hydrolyzed by ubiquitin C-terminal hydrolase L3 (UCH-L3). FEBS Lett. 585:2568-2574

5. Drorbaugh JE, Fenn WO (1955) A barometric method for measuring ventilation in newborn infants. Pediatrics 16:81-87

6. Drozdz A (1975) Food habits and food assimilation in mammals. In: Grodzinski W, Klekowski RZ, Duncan A (eds) Methods for Ecological Bioenergetics. Blackwell Sci. Pub. Oxford, UK

7. Fischer DF, van Dijk R, van Tijn P, Hobo B, Verhage MC, van der Schors RC, Li KW, van Minnen J, Hol EM, van Leeuwen FW (2009) Long-term proteasome dysfunction in the mouse brain by expression of aberrant ubiquitin. Neurobiol. Aging 30:847-863

8. Fuchs H, Gailus-Durner V, Adler T, Aguilar-Pimentel JA, Becker L, Calzada-Wack J, Da Silva-Buttkus P, Neff F, Gotz A, Hans W, et al. (2011) Mouse phenotyping. Methods 53:120-135

9. Gailus-Durner V, Fuchs H, Adler T, Aguilar Pimentel A, Becker L, Bolle I, Calzada-Wack J, Dalke C, Ehrhardt N, Ferwagner B, et al. (2009) Systemic first-line phenotyping. Methods Mol. Biol. 530:463-509

10. Hope AD, de Silva R, Fischer DF, Hol EM, van Leeuwen FW, Lees AJ (2003) Alzheimer's associated variant ubiquitin causes inhibition of the 26S proteasome and chaperone expression. J. Neurochem. 86:394-404

11. Kallnik M, Elvert R, Ehrhardt N, Kissling D, Mahabir E, Welzl G, Faus-Kessler T, de Angelis MH, Wurst W, Schmidt J, et al. (2007) Impact of IVC housing on emotionality and fear learning in male C3HeB/FeJ and C57BL/6J mice. Mamm. Genome 18:173-186

12. King DL, Arendash GW, Crawford F, Sterk T, Menendez J, Mullan MJ (1999) Progressive and gender-dependent cognitive impairment in the APP(SW) transgenic mouse model for Alzheimer's disease. Behav. Brain Res. 103:145-162

13. Knight EM, Verkhratsky A, Luckman SM, Allan SM, Lawrence CB (2012) Hypermetabolism in a triple-transgenic mouse model of Alzheimer's disease. Neurobiol. Aging 33:187-189

14. Ko S, Kang GB, Song SM, Lee JG, Shin DY, Yun JH, Sheng Y, Cheong C, Jeon YH, Jung YK, et al. (2010) Structural basis of E2-25K/UBB+1 interaction leading to proteasome inhibition and neurotoxicity. J. Biol. Chem. 285:36070-36080

15. Lee J, Lee Y, Lee MJ, Park E, Kang SH, Chung CH, Lee KH, Kim K (2008) Dual modification of BMAL1 by SUMO2/3 and ubiquitin promotes circadian activation of the CLOCK/BMAL1 complex. Mol. Cell. Biol. 28:6056-6065

16. Lewandowski NM, Small SA (2005) Brain microarray: finding needles in molecular haystacks. J. Neurosci. 25:10341-10346

17. Lindecke A, Korte M, Zagrebelsky M, Horejschi V, Elvers M, Widera D, Prullage M, Pfeiffer J, Kaltschmidt B, Kaltschmidt C (2006) Long-term depression activates transcription of immediate early transcription factor genes: involvement of serum response factor/Elk-1. Eur. J. Neurosci. 24:555-563

18. Nakashiba T, Buhl DL, McHugh TJ, Tonegawa S (2009) Hippocampal CA3 output is crucial for ripple-associated reactivation and consolidation of memory. Neuron 62:781-787

19. Ohl F, Sillaber I, Binder E, Keck ME, Holsboer F (2001) Differential analysis of behavior and diazepam-induced alterations in C57BL/6N and BALB/c mice using the modified hole board test. J. Psychiatr. Res. 35:147-154

20. Park Y, Yoon SK, Yoon JB (2009) The HECT domain of TRIP12 ubiquitinates substrates of the ubiquitin fusion degradation pathway. J. Biol. Chem. 284:1540-1549

21. Prinzen C, Trumbach D, Wurst W, Endres K, Postina R, Fahrenholz F (2009) Differential gene expression in ADAM10 and mutant ADAM10 transgenic mice. BMC Genomics 10:66

22. Rainer J, Sanchez-Cabo F, Stocker G, Sturn A, Trajanoski Z (2006) CARMAweb: comprehensive R- and bioconductor-based web service for microarray data analysis. Nucleic Acids Res. 34:W498-503

23. Ramakers C, Ruijter JM, Deprez RH, Moorman AF (2003) Assumption-free analysis of quantitative real-time polymerase chain reaction (PCR) data. Neurosci. Lett. 339:62-66

24. Reinhard C, Eder G, Fuchs H, Ziesenis A, Heyder J, Schulz H (2002) Inbred strain variation in lung function. Mamm. Genome 13:429-437

25. Ryu KY, Fujiki N, Kazantzis M, Garza JC, Bouley DM, Stahl A, Lu XY, Nishino S, Kopito RR (2010) Loss of polyubiquitin gene Ubb leads to metabolic and sleep abnormalities in mice. Neuropathol. Appl. Neurobiol. 36:285-299

26. Schonberger SJ, Edgar PF, Kydd R, Faull RL, Cooper GJ (2001) Proteomic analysis of the brain in Alzheimer's disease: molecular phenotype of a complex disease process. Proteomics 1:1519-1528

27. Schulz H, Johner C, Eder G, Ziesenis A, Reitmeier P, Heyder J, Balling R (2002) Respiratory mechanics in mice: strain and sex specific differences. Acta Physiol. Scand. 174:367-375

28. Tankersley CG (2003) Genetic aspects of breathing: on interactions between hypercapnia and hypoxia. Respir. Physiol. Neurobiol. 135:167-178

29. Tilleman K, Van den Haute C, Geerts H, van Leuven F, Esmans EL, Moens L (2002) Proteomics analysis of the neurodegeneration in the brain of tau transgenic mice. Proteomics 2:656-665

30. Untergasser A, Nijveen H, Rao X, Bisseling T, Geurts R, Leunissen JA (2007) Primer3Plus, an enhanced web interface to Primer3. Nucleic Acids Res. 35:W71-74

31. Van Tijn P, Hobo B, Verhage MC, Oitzl MS, van Leeuwen FW, Fischer DF (2011) Alzheimer-associated mutant ubiquitin impairs spatial reference memory. Physiol. Behav. 102:193-200

32. Vandesompele J, De Preter K, Pattyn F, Poppe B, Van Roy N, De Paepe A, Speleman F (2002) Accurate normalization of real-time quantitative RT-PCR data by geometric averaging of multiple internal control genes. Genome Biol. 3:R34.I-II

33. Wang H, Jia N, Fei E, Wang Z, Liu C, Zhang T, Fan J, Wu M, Chen L, Nukina N, et al. (2007) p45, an ATPase subunit of the 19S proteasome, targets the polyglutamine disease protein ataxin-3 to the proteasome. J. Neurochem. 101:1651-1661

34. Yano M, Nakamuta S, Wu X, Okumura Y, Kido H (2006) A novel function of 14-3-3 protein: 14-3-3zeta is a heat-shock-related molecular chaperone that dissolves thermal-aggregated proteins. Mol. Biol. Cell 17:4769-4779
